# Integrating InVEST and machine learning to model mangrove habitat degradation trend in in Northern Persian Gulf, Iran

**DOI:** 10.1038/s41598-026-47305-z

**Published:** 2026-04-21

**Authors:** Mohamad Kazemi, Atefeh Jafarpoor, Youssef Ahmadi, Raoof Mostafazadeh

**Affiliations:** 1 Department of Hormoz Studies and Research Center, University of Hormozgan, Bandar Abbas, Iran; 2 Department of Natural Resources and Environment, Faculty of Agriculture, Shahid Bahonar University of Kerman, Kerman, Iran; 3 Department of Natural Resources and member of Water Management research center, Faculty of Agriculture and Natural Resources, University of Mohaghegh Ardabili, Ardabil, Iran

**Keywords:** Mangrove degradation, Remote sensing, Enhanced mangrove vegetation index, Machine learning models, Climate sciences, Ecology, Ecology, Environmental sciences, Natural hazards

## Abstract

The expansion of human activities and environmental changes in coastal desert and semi-desert ecosystems significantly impact habitat quality. This study used remote sensing data on the Google Earth Engine (GEE) platform to generate layers representing threats to mangrove ecosystems, including human construction, runoff, maximum daily temperature, wind speed, access roads, population density, drought severity, NO₂, SO₂, land surface temperature, soil moisture deficiency, actual evapotranspiration, and dust. These layers were analyzed for current and future conditions, and a target layer for mangrove change detection was created using a variable sampling method and the Random Forest algorithm for both time periods. The Mangrove Vegetation Index (MVI) and Enhanced Mangrove Vegetation Index (EMVI) were included as auxiliary data. The resulting data were analyzed using the InVEST model to generate maps of habitat quality and mangrove degradation, and habitat quality was further assessed using machine learning models. The results showed that Linear Trend, Neural Network and SVR models performed better than other machine learning models. Also, in the study area, habitat quality will decrease according to the Otsu threshold in the future years. The results reveal that, under current conditions, 35.43% of the mangrove forest area is classified as experiencing low-intensity degradation. Projections indicate that, under future conditions, 33.33% of the area will shift to high-intensity degradation, especially in southern regions near Qeshm Island. Habitat quality is also to decline, with the “good and suitable” habitat quality category expected to decrease by 3.52%. Additionally, human activity indices in medium and high categories are projected to increase by 5.38 km² and 4.48 km², respectively. These results can be used to guide future coastal management and conservation strategies by identifying areas at highest risk of mangrove degradation and declining habitat quality under increasing human pressure and environmental change.

**Supplementary Information:**

The online version contains supplementary material available at 10.1038/s41598-026-47305-z.

## Introduction

Mangrove forests, composed of woody plants, thrive in the intertidal zones between land and sea in tropical and subtropical regions worldwide^1,2^. Renowned for their diverse ecological services and positive environmental impacts, mangroves are recognized as unique coastal habitats and sensitive marine ecosystems. Consequently, many areas manage these habitats as protected zones^3,4^. Due to their intricate structure, high species diversity, substantial productivity, specific ecological functions, and significant socio-economic value, mangrove forests are highly esteemed^5^. However, these ecosystems are under serious threat from human activities, including overexploitation, urban development, rising sea levels, and excessive logging^4,6^. These pressures lead to biodiversity loss and degradation of essential ecosystem services^6^. Given these mounting pressures, effective conservation requires reliable and spatially explicit monitoring tools. While previous studies have primarily assessed mangrove habitat quality using static land-use categories or general vegetation indices, they often lack the integration of mangrove-specific spectral indices (e.g., MVI, EMVI) with dynamic threat layers and machine learning-based projections to capture the nuanced degradation trends in these unique coastal ecosystems. Thus, evaluating habitat quality in mangroves through such an integrated approach is critical for conservation and restoration efforts.

Thus, evaluating habitat quality in mangroves is critical for conservation and restoration efforts. Habitat quality indicates an ecosystem’s ability to provide the resources and conditions necessary for wildlife survival. It plays a crucial role in determining biodiversity^7^. It encompasses the environmental conditions essential for the survival and populations of living organisms, represented as a numerical variable that ranges from low to high^8,9^. Higher habitat quality correlates with a more stable ecological structure and function within the ecosystem^10–12^. In this context, researchers have extensively employed the Integrated Valuation of Ecosystem Services and Tradeoffs (InVEST) model to quantify habitat quality^7,13–15^. The InVEST model is distinguished by its quantitative accuracy, result visualization, and cost-effectiveness, which contribute to its widespread application in studies assessing ecosystem service performance^16^. Overall, it is considered one of the most effective tools for evaluating habitat quality. By utilizing land-use change data and identifying biodiversity threats, the model generates habitat quality maps and provide valuable indications for conservation planning and forecasting future changes over time^17^. While InVEST has proven effective in various terrestrial landscapes, its application in complex coastal ecosystems like mangroves remains less explored, partly due to challenges in accurately mapping mangrove extent and health.

While research on habitat quality has yielded initial results, most studies have concentrated on protected areas, watersheds, and urban regions, with comparatively fewer investigations focused on mangrove forests^18^. As vital wetland reserves, these forests require robust ecological security and effective biodiversity conservation. Conversely, human activities (such as deforestation, urbanization, road construction, industrial operations, mining, and climate change) have severely impacted natural habitats. Habitat degradation is recognized as a primary driver of biodiversity loss and a decline in species abundance. However, the intensity of degradation varies in its effects on habitat quality^19,20^. Thus, understanding the key factors that influence habitat quality and degradation, particularly in mangrove forests, is essential for identifying existing threats and enhancing overall habitat quality. However, an integrated framework that combines these advanced mapping techniques with the habitat assessment capability of InVEST and the predictive power of machine learning for mangrove ecosystems is still lacking.

In this context, remote sensing technology has emerged as a valuable tool for mapping mangrove forests^4,21^ and assessing habitat quality. Various vegetation indices have been utilized for vegetation mapping, including the Normalized Difference Vegetation Index (NDVI)^22,23^. However, these indices are not tailored for mangroves and often struggle to differentiate mangroves from terrestrial vegetation. To address this limitation, some researchers have developed specialized indices for extracting mangrove data using different satellite data input bands, such as the Mangrove Vegetation Index (MVI) and the Enhanced Mangrove Vegetation Index (EMVI)^6^. The Mangrove Vegetation Index (MVI), created using the NIR, SWIR1, and green bands from Sentinel-2 satellites, offers a straightforward and efficient method for classifying mangrove forests^6^. MVI takes into account the unique greenness and moisture content characteristics of mangrove vegetation, enabling effective differentiation between mangroves and non-mangrove areas, such as water, soil, and developed lands^4^.

On the other hand, the use of machine learning has been used in assessing habitat quality^24^. In this regard, Saoum and Sarkar^25^ announced in a comprehensive assessment of changes in the mangrove forests of the Sundarbans region in the years 2004 to 2022 that there is a decreasing trend in mangrove forests at a rate of 2.66% per year in this region and their prediction until 2030 is also based on the decreasing trend in mangrove forests. Also, the research of Farazmansh et al.^26^ using Landsat data from 2000 to 2022 and using an artificial neural network algorithm, predicted changes in mangrove forests in two protected areas in the southwest Madagascar and Abu Dhabi, United Arab Emirates for the years 2023 to 2027. Their results showed that there was a fluctuating trend in the area of the studied mangrove forests and a gradual improvement was seen at the end of the mangrove forest protection periods. Naseri et al.^27^ investigated habitat quality changes in Karaj County, Iran, from 2013 to 2032. Using Landsat imagery, LULC classification, and the InVEST model with CA-Markov predictions, they assessed the impact of land use and threats such as agriculture, urban areas, and roads^28^.

Accurately mapping mangrove extent is a critical first step for any habitat assessment. Traditional vegetation indices like NDVI often fail to distinguish mangroves from other vegetation. Therefore, specialized indices such as the Mangrove Vegetation Index (MVI) and Enhanced Mangrove Vegetation Index (EMVI) have been developed for more reliable detection using satellite data like Sentinel-2. Concurrently, machine learning (ML) algorithms, particularly Random Forest, have become pivotal in classifying land cover and predicting ecological trends. Despite these advances, a comprehensive approach that integrates high-accuracy mangrove mapping (using GEE and specialized indices), habitat quality modeling (via InVEST), and predictive forecasting (using ML) for mangrove conservation is not well established.

However, methodological limitations persist in mangrove habitat quality assessments. Many studies rely on generic threat variables and broad land-use classifications not tailored to mangrove ecosystems’ unique sensitivities. Furthermore, there is a scarcity of integrated approaches that dynamically combine satellite-derived, ecosystem-specific indices (e.g., MVI, EMVI) with a comprehensive suite of bioclimatic and anthropogenic threat layers within a spatially explicit modeling framework like InVEST, followed by robust machine learning forecasting to predict future degradation trends. This study aims to bridge this gap by developing a novel, integrated assessment framework specifically for mangrove forests.

While the aforementioned machine learning studies effectively demonstrate the utility of algorithms like ANN and Random Forest for detecting and projecting changes in mangrove extent, they primarily address areal loss or gain. However, the critical next step for conservation is understanding the qualitative change within these habitats, their degradation and quality^29–31^. This study bridges this gap by building upon the established capability of machine learning to model complex spatial-temporal patterns. We advance the application by using machine learning not for area change detection, but to predict future habitat quality scores derived from the InVEST model. This represents a significant conceptual shift from monitoring quantity to forecasting ecological condition, enabling a more nuanced assessment of mangrove ecosystem vulnerability. Therefore, this research integrates the methodological strengths of prior machine learning applications with a novel focus on habitat quality prediction, addressing a key need for proactive management in the face of environmental change.

While MVI and EMVI have been successfully applied for mangrove detection in standalone studies [e.g., 4, 6], and variable/migrated sampling methods are known in change detection literature, their integrated application within a GEE-InVEST-ML framework for habitat quality assessment is novel. Specifically, prior InVEST-based habitat studies in mangroves often rely on coarse or static land-cover inputs. Our innovation lies in leveraging these advanced remote sensing techniques to generate high-accuracy, multi-temporal LULC maps specifically optimized for mangroves, which then serve as the primary dynamic input for the InVEST model. This approach directly addresses a key limitation: it enhances the temporal sensitivity and ecological relevance of the habitat quality model by ensuring the foundational land-cover data accurately reflects mangrove-specific phenology and changes, leading to more reliable degradation and quality maps compared to using generic or outdated LULC products.

This study mapped mangrove forests for 2019, 2022, and 2024 using Sentinel-2 imagery on Google Earth Engine (GEE). The mapping combined mangrove-specific spectral indices with auxiliary data and applied the Random Forest algorithm, incorporating a variable sampling method for training data generation. Satellite-derived data on population density, wind speed, dust, and other environmental threats were integrated as raster inputs into the InVEST model. The results support sustainable land management, conservation planning, and mitigation of human impacts on mangroves, while providing a foundation for future ecosystem service monitoring in the region. Key innovations include: (1) applying the migrated samples method within GEE to produce LULC maps as an input for mangrove identification; (2) using auxiliary indices (e.g., MVI, EMVI) from Sentinel-2 to detect ecosystem changes; and (3) integrating satellite-derived environmental threat variables into the InVEST model for robust habitat quality and degradation assessments.

Regarding the significance of this research it can be said that this study presents a novel, integrated GEE-InVEST-Machine Learning framework for dynamic mangrove monitoring. Its methodological novelty is enhanced by employing variable sampling and specialized mangrove indices (MVI, EMVI). As a vital ecosystem in the Persian Gulf, the degradation quantified here serves as an early warning for policymakers. The results provide a scientific tool for conservation planning by pinpointing critical zones and forecasting trends. The integrated approach can serve as a model for monitoring other mangrove ecosystems globally under increasing anthropogenic and environmental pressures.

The aim of the current research was (a) to assess and analyze the spatio-temporal habitat quality and degradation of mangrove forests in the Khorkhoran protected area using the InVEST model. (b) To integrate multiple threat layers (including anthropogenic, climatic, and environmental factors) derived from the Google Earth Engine platform into the habitat quality assessment, (c) to predict future changes in habitat quality and degradation intensity by employing machine learning methods and comparing model performance, (d) to identify critical and high-risk zones within the mangrove forests in terms of degradation and habitat quality decline for targeted management strategies.

## Materials and methods

### Study area

**Fig. 1:**
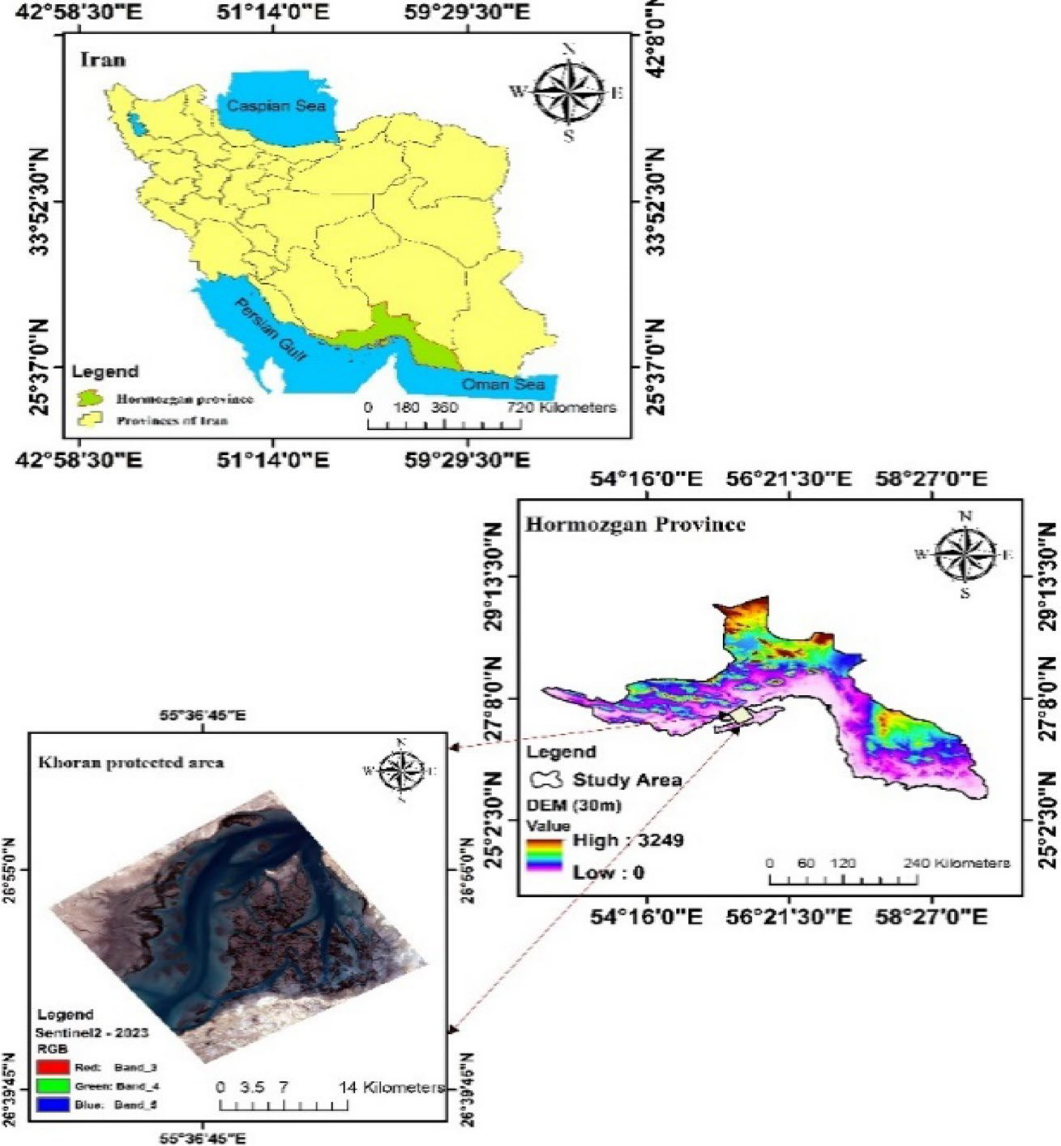
Location of the studied area (Khorkhoran mangrove forests) in Iran and Hormozgan province (Map processing and creation were carried out using ArcMap within ArcGIS version 10.1^32^.

The study area is the Khorkhoran Mangrove Forest Protected Area, located in Hormozgan Province, Iran. As illustrated in Fig. 1, this region stretches along the southern coastline from Khamir City to the villages of Laft and Tabl on Qeshm Island. It is located between 43°26’–59°26’ N latitude and 27°55’–48°55’ E longitude.

The Khorkhoran International Wetland and its Hara (mangrove) forests are major tourist attractions in Hormozgan Province and the port city of Khamir. Ecotourism activities such as boating, fishing, wildlife observation, and local homestays provide direct and indirect income for local communities. The mangrove habitat has saline, halomorphic soils formed by seasonal river sediments, rich in fine particles, organic matter, and decayed plant materials. The area includes mudflats, mangrove forests, salt marshes, riverine and marine coastal habitats. Its unique ecosystem supports high biodiversity, especially migratory waterbirds and marine species. The dominant tree species is Avicennia marina, along with herbaceous halophytes. The region has a hot desert climate, with an average annual rainfall of about 200 mm and temperatures between 26 and 27 °C. July is the hottest month (34 °C) and January the coldest (18 °C). Due to its ecological importance, the wetland and Hara forests are registered as a UNESCO Biosphere Reserve. Among several mangrove sites in Hormozgan, the Khorkhoran mangrove forest in Bandar Khamir is the largest marine forest in the Middle East.

### Data preparation

In the current study, a variable sampling method and the Random Forest classification algorithm were employed to generate land use maps for the target years 2019, 2022, and 2024. Sentinel-2 Level-1 C Top-Of-Atmosphere (TOA) reflectance images were used. The specific image acquisition dates were selected to coincide with the peak phenological stage of the mangroves: imagery from the first quarters (January-March) of 2019 and 2024 were used. The images are geometrically corrected to an orthorectified coordinate system and have already undergone radiometric correction. Topographic effects are automatically adjusted during orthorectification processing. It should be noted that the study area in the present research is flat and plain, eliminating the need for topographic shadow removal. Sentinel-2 data are widely used for various applications, such as classifications, vegetation indices, and qualitative analyses^33^, featuring 13 spectral bands, were accessed through the Google Earth Engine (GEE) platform. The bands utilized in this study included 2, 3, 4, 7, 8, 11, and 12, corresponding to blue, green, red, near-infrared (NIR), shortwave infrared 1 (SWIR1), shortwave infrared 2 (SWIR2), and red-edge (RE) bands. All bands were resampled to a spatial resolution of 10 m, mosaicked, and averaged to prepare land use maps.

Additionally, the European Space Agency’s (ESA/WorldCover/v100) land use map, with a spatial resolution of 10 m, was used to generate reference training data^34^. The study defined six land use classes based on an operational remote sensing classification: mangrove forests (code 0), non-mangrove wetlands including intertidal mudflats and open water bodies (code 1), salt marshes and barren lands (code 2), residential and urban areas (code 3), pastures and agricultural lands (code 4), and vegetated wetlands such as reed beds (code 5). It should be noted that this classification represents a remote-sensing-based distinction optimized for mangrove mapping rather than a strict ecological separation of all wetland types. The final land-use/land-cover (LULC) maps produced for the years 2019, 2022, and 2024 served as primary inputs for the subsequent habitat quality and degradation analysis using the InVEST model.

The classification scheme distinguishes ecologically and functionally distinct units within the Khorkhoran wetland system to enable accurate habitat quality assessment. Although the study area represents a wetland complex, the mangrove forests class (0) specifically includes areas dominated by Avicennia marina, while the wetlands class (1) represents open water and permanently flooded areas without emergent vegetation. The wet wetlands class (5) includes other vegetated, frequently inundated areas (e.g., reed beds or salt marshes) distinct from mangroves, allowing the InVEST model to assign habitat suitability and assess threats to mangroves separately from adjacent non-mangrove wetlands.

To address scale conflicts between input threat layers (e.g., population density in people/km², temperature in °C, pollutant concentrations in mg/m³), all raster layers were normalized to a common dimensionless scale of 0 to 1 before integration into the InVEST model using min-max scaling. This ensures proportional contributions to threat intensity without bias from original units. Consequently, the relative weight (Wₗ) assigned to each threat (Table 1) operates on a uniform scale, enabling a balanced assessment of cumulative habitat impacts.

It is noteworthy that Sentinel-2 satellite images were selected to align with the phenological growth stages of mangrove forests in the study area. In addition to basic pixel classification using spectral bands, spectral indices were incorporated as auxiliary data to enhance the classification process. Sentinel-2 data were specifically acquired during the first quarters of 2019 and 2024 to capture the annual phenological peak when mangroves exhibit their highest greenness values in the study area. The spectral indices used were as follows:

#### Mangrove vegetation index (MVI)

Given the significant presence of mangrove forests in the study area, the Mangrove Vegetation Index (MVI) was employed to map the extent of mangroves using remote sensing images. The calculation of the MVI is defined by the equation shown in Eq. ( 1)^4,6^.

1 $$\:MVI=\frac{NIR-Green}{SWIR1-Green}$$

#### Enhanced mangrove vegetation index (EMVI)

To more accurately assess mangrove vegetation in the study area, the Enhanced Mangrove Vegetation Index (EMVI) was utilized. This index is based on the green and shortwave infrared (SWIR) bands, enabling the detection of differences in canopy greenness and moisture between mangrove and non-mangrove vegetation. The formula for EMVI is presented in Eq. (2)^6,35^.

2 $$\:EMVI=\frac{\mathrm{G}\mathrm{r}\mathrm{e}\mathrm{e}\mathrm{n}-\mathrm{S}\mathrm{W}\mathrm{I}\mathrm{R}2}{\mathrm{S}\mathrm{W}\mathrm{I}\mathrm{R}1-\mathrm{G}\mathrm{r}\mathrm{e}\mathrm{e}\mathrm{n}}$$

As mentioned, the initial training samples for the year 2019 were derived from the land use map provided by the European Space Agency. In the next step, variable training samples for the years 2022 and 2024 were calculated using the Spectral Angle Distance (SAD) method. This method was employed to quantify the difference between reference and target spectra, helping to identify consistent training samples. The formulas for the SAD index are presented in Eqs. (4, 5)^36,37^.

3 $$\:SAD=\:\mathrm{cos}\theta\:$$

4 $$\:\theta\:={cos}^{-1}\frac{\sum\:_{i=1}^{N}{A}_{i\left(t1\right){B}_{i\left(t2\right)}}}{\sqrt{\sum\:_{i=1}^{N}{\left({A}_{i\left(t1\right)}\right)}^{2}\sum\:_{i=1}^{N}{\left({B}_{i\left(t1\right)}\right)}^{2}}}$$

Where θ is the spectral angle, *A* represents the reference spectrum at time *t*, *B* is the spectrum at time *t*2, *i* is the band index, and *N* is the number of bands. The Spectral Angle Distance (SAD) ranges between 0 and 1^37^. When the reference and target spectra are identical, the SAD value equals 1.

In the subsequent step, 70% of the samples were used for training, while the remaining 30% were allocated for testing, evaluation, and accuracy assessment of the generated maps^38^. The Random Forest (RF) machine learning algorithm was then employed to classify the images. RF is one of the most robust learning algorithms, originally developed by Leo Breiman and Adele Cutler. It enhances traditional tree-based regression and classification models^39,40^.

The RF algorithm is based on an ensemble learning method that constructs numerous decision trees and combines their results to enhance prediction accuracy. By aggregating multiple decision trees, this method reduces model sensitivity and instability, thereby improving robustness and accuracy. Each decision tree in the RF model classifies input vectors independently, with the final output determined by majority voting of the class labels. The trees are constructed using the bootstrap method, with a random subset of input variables selected for each tree to minimize correlation among them.

Key advantages of the RF algorithm include reducing model inconsistency, increasing predictive performance, and eliminating the need for additional calibration^39,41^. To assess the accuracy of the land use maps, a confusion matrix was employed to calculate overall accuracy, Kappa coefficient, producer’s accuracy, user’s accuracy, commission error, and omission error^42^. Due to logistical constraints and the inaccessibility of parts of the protected wetland, direct field-based ground truthing for the classification years (2022, 2024) was not feasible. To ensure classification reliability, we employed a robust alternative strategy. First, the high-resolution (10 m) and globally validated ESA WorldCover product for 2019 served as the foundational reference for initial training samples. Second, the variable sampling method using Spectral Angle Distance ensured temporal consistency of these samples for subsequent years. Third, the classification accuracy was rigorously quantified using standard metrics (Overall Accuracy, Kappa) derived from a statistically independent test set (30% of samples). While field validation would further strengthen the results, this multi-layered approach utilizing trusted reference data, temporal sample transfer, and quantitative accuracy assessment provides a credible and reproducible basis for the land use maps used in the subsequent InVEST modeling.

**Fig. 2:**
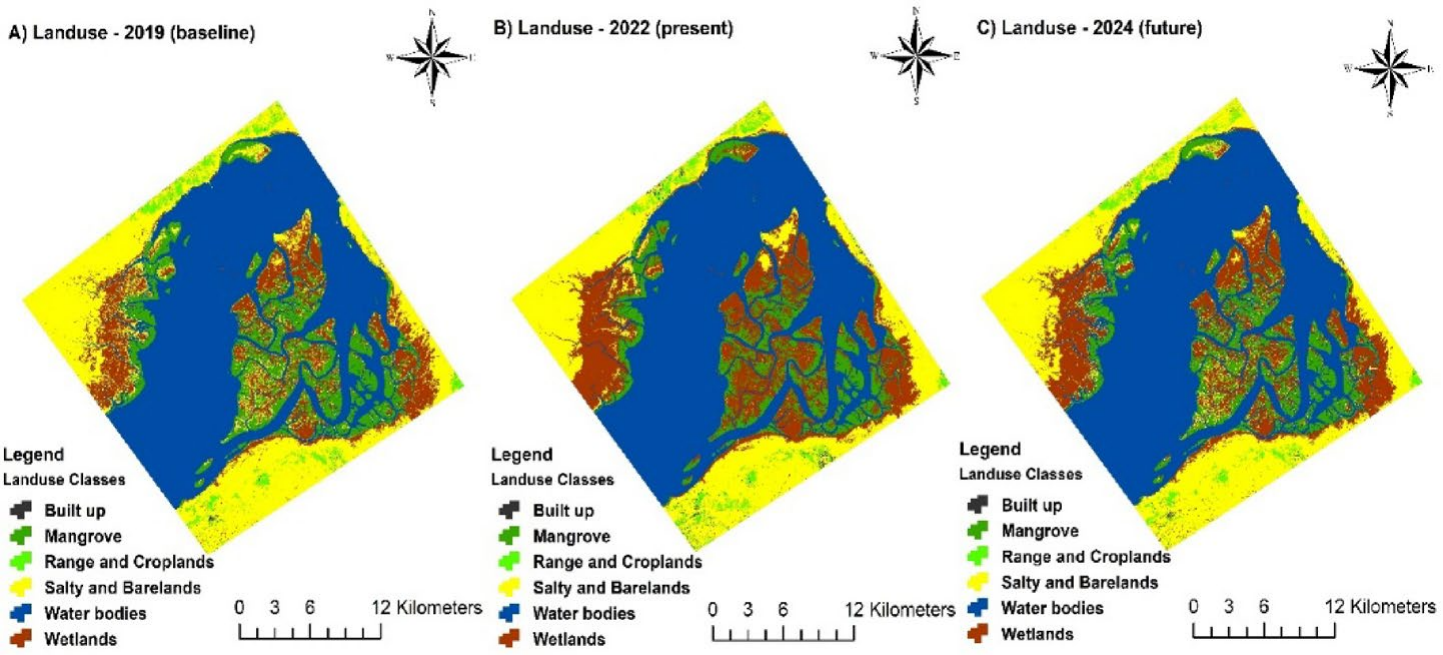
Land use map in the studied times (Map processing and creation were carried out using ArcMap within ArcGIS version 10.1^32^.

After preparing the dependent variables, all independent variables were incorporated as potential threats to mangroves on the Google Earth Engine (GEE) platform^43–45^. The spatial maps of these variables for both current and future scenarios are presented in Fig. 2.

### The invest habitat quality model

In the current study, the Integrated Valuation of Ecosystem Services and Trade-offs (InVEST) model, a habitat-based assessment tool, was utilized to calculate the quality and degradation of mangrove forests in the Khorkhoran region. The model integrates data on land use across different years and sources of threats to mangrove habitats, generating maps that depict habitat quality and degradation. Mangrove species with varying degrees of suitability in land use maps and their sensitivity to different threats were considered in the analysis.

In the first step, land use maps for the baseline, current, and future years were prepared^14,16^. Mangrove forests were assigned a value of 1 (good habitat), while other land uses were coded as 0 (non-habitat)^16^. This methodology is rooted in the island-ocean model, which assumes that a managed matrix surrounding natural, unmanaged patches makes them unsuitable as habitat from a species’ perspective^46^.

In the second step, the model incorporated data on habitat threats, including their severity and impacts on habitat quality. The effects of these threats were analyzed within a raster framework, where the relative impact of each threat “representing its intensity” was assigned a value between 0 and 1. Additionally, the distance between the habitat and the source of the threat was factored in as a mitigating variable.

The model also accounted for the level of legal protection in each pixel, represented as a reduction factor. This protection level was assigned a value between 0 and 1, where 1 signifies complete access of threats to the conserved area, and 0 indicates no access. In this study, a value of 0.5 was assigned, reflecting 50% efficiency of the conserved area in mitigating threats from land use changes.

The final factor in calculating habitat degradation is habitat sensitivity to each threat. This sensitivity, denoted as *Sjr*​, measures the vulnerability of habitat type *j* to threat *r* and is assigned a value between 0 and 1, with higher values indicating greater sensitivity^47,48^. The model assumes that higher sensitivity results in greater habitat degradation^49,50^.

In the final step, the relative sensitivity of each habitat type to each threat was calculated. The overall threat level for pixel x with land use or habitat type j, denoted as Dxj​, is computed using Eq. 5. The model’s assumption that greater sensitivity corresponds to higher habitat degradation is supported by prior studies^13^.

4 $$\:{D}_{xj}=\sum\:_{r=1}^{R}\sum\:_{r=1}^{{Y}_{r}}\left(\frac{{W}_{r}}{\sum\:_{r=1}^{R}{W}_{r}}\right){r}_{y}{i}_{rxy}{\beta\:}_{x}{S}_{jr}$$

Where D_xj_ is the overall threat in pixel x with habitat type j, R represents the total number of threat factors, and Y_r_ is the set of pixels associated with threat map r. W_r_ denotes the relative importance of each threat compared to others. The variable r_y_ represents the effect of threat factor r originating from pixel y, which impacts the habitat in pixel x as i_rxy_. Additionally, β_x_ corresponds to the accessibility level of pixel x, while S_jr_ indicates the relative sensitivity of each habitat type j to the corresponding threat r.

In this equation, if S_jr_=0, then D_xj_ is not influenced by threat r. The variable i_rxy_ represents the value of the threat source r_y_ in the network y, which can affect the habitat either linearly or exponentially, as described in Eqs. 6 and 7^11^.

6 $$\:{i}_{rxy}=\left\{1-\left(\frac{{d}_{xy}}{{d}_{r\:max}}\right)\right\}\:\:\:\:\left(\mathrm{L}\mathrm{i}\mathrm{n}\mathrm{e}\mathrm{a}\mathrm{r}\:\mathrm{d}\mathrm{e}\mathrm{c}\mathrm{a}\mathrm{y}\right)$$

7 $$\:{i}_{rxy}=exp\left[-\left(\frac{2.99}{{d}_{r\:max}}\right){d}_{xy}\right]\:\:\:\:\:\:\left(\mathrm{E}\mathrm{x}\mathrm{p}\mathrm{o}\mathrm{n}\mathrm{e}\mathrm{n}\mathrm{t}\mathrm{i}\mathrm{a}\mathrm{l}\:\mathrm{d}\mathrm{e}\mathrm{c}\mathrm{l}\mathrm{i}\mathrm{n}\mathrm{e}\right)$$

Where d_xy_ represents the distance between pixels and the effects of threats on habitat quality. Generally, the longer the distance, the lower the impact of the threat source on the habitat. d(r_max_) denotes the maximum distance of the threat factor’s influence on the habitat. This value defines the distance beyond which the threat source’s impact is minimized and no longer significantly affects the habitat.

In the final step of model execution, the degradation score of a cell is calculated using the half-saturation function (Eq. 8). This function converts the degree of pixel degradation into habitat quality. As the degradation level increases, habitat quality decreases. The habitat quality in pixel *x*, located in land use class *j*, is denoted by *Qxj*​ ^49^.

8 $$\:{Q}_{xj}={H}_{j}\left(1-\left(\frac{{D}_{{x}^{j}}^{z}}{{D}_{xj}^{z}+{K}^{z}}\right)\right)$$

**Fig. 3:**
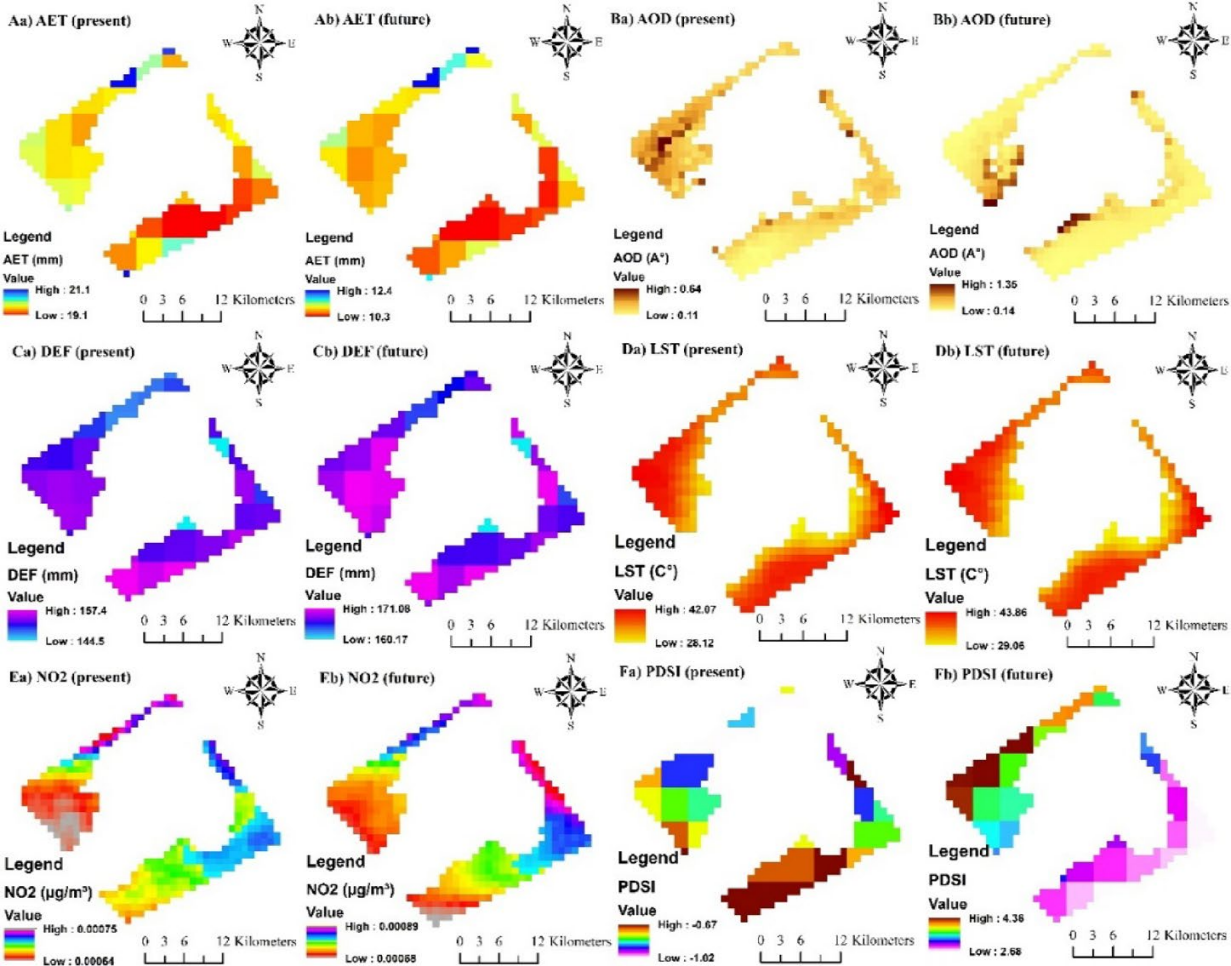
Khorkhoran mangrove threat map from the google earth engine (for the present and the future (Map processing and creation were carried out using ArcMap within ArcGIS version 10.1^32^.

**Fig. 4:**
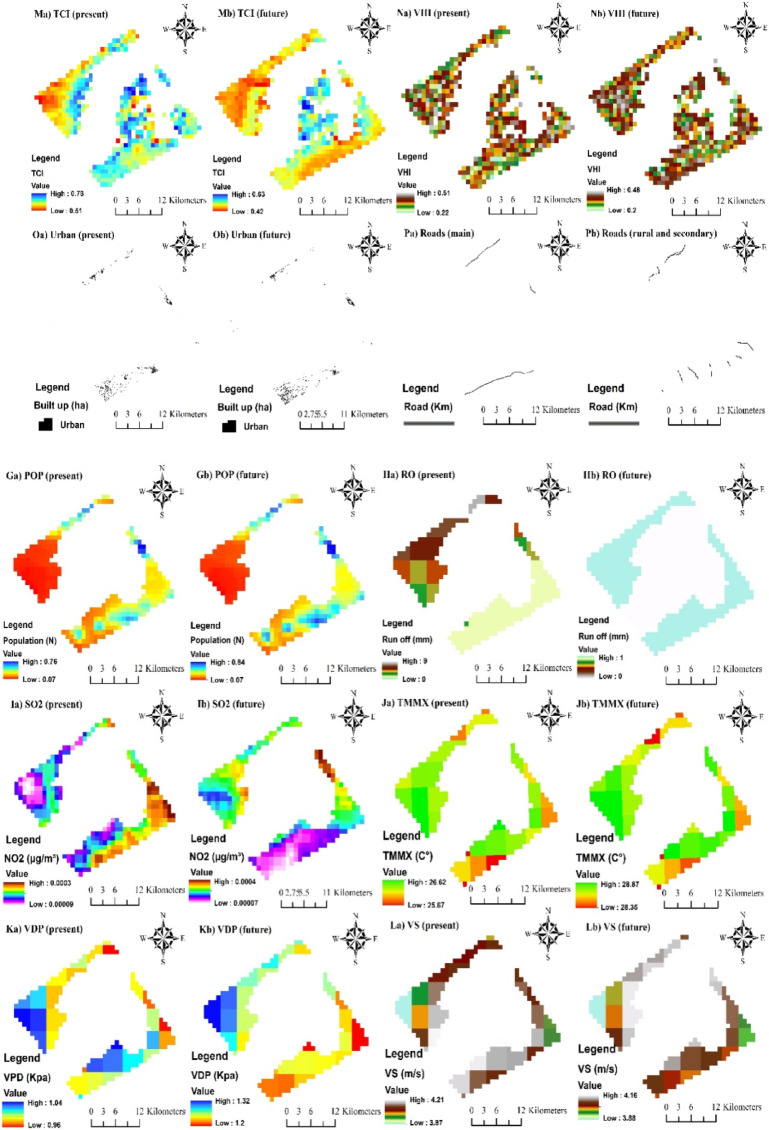
(cont.). Khorkhoran mangrove threat map from the google earth engine (for the present and the future (Map processing and creation were carried out using ArcMap within ArcGIS version 10.1^32^.

In this context, *Qxj* represents the habitat quality of pixel x*x* in land use *j*, *Hj* is the habitat desirability in land use *j*, and *D*(*xj*)*z* is the habitat threat level for pixel *x* in land use *j*. *K* is the constant semi-saturation or saturation coefficient, which is half the maximum value of *Dxj*, and *z* is a constant with a value of 2.5. If *Hj* = 0, then *Qxj* will be zero. *Qxj* increases with *Hj* and decreases with *D*(*xj*)*z*, with its value capped at a maximum of 1 ^17^. In Figs. 3, and 4 the threat maps taken from the Google Earth Engine (GEE) is presented for both the present and future scenarios.

On the other hand, in the current study, the impact range, weight, and sensitivity of the mangrove habitat to each threat, along with other parameters, were determined based on the InVEST software guidelines and expert opinions, as outlined in Table 1.

The threat parameter values in Table 1 (MAX_DIST, WEIGHT, DECAY, sensitivity) were derived from a synthesis of: (1) the official InVEST model guidelines, (2) a review of analogous mangrove/coastal studies [e.g., 4, 6], and (3) a structured Delphi survey with ten experts in mangrove ecology, remote sensing, and coastal management. All threat raster layers were sourced from Google Earth Engine using established global datasets, including climate variables from ERA5-Land and TerraClimate (e.g., temperature, evapotranspiration, soil moisture, wind, and the PDSI drought index), pollution data from Sentinel-5P and MODIS (NO₂, SO₂, AOD), anthropogenic factors from WorldPop, OpenStreetMap, and ESA WorldCover (population density, roads, urban areas), and mangrove-specific indices (MVI, EMVI) from Sentinel-2 imagery. Preliminary parameters were set from literature and InVEST documentation. These were refined via a two-round expert Delphi survey to calibrate weights and influence distances to the specific conditions of the Khorkhoran mangroves (Persian Gulf), enhancing local model relevance. Decay functions (linear/exponential) were assigned based on threat nature (e.g., exponential for point-source pollution, linear for road access).

It is important to clarify that variables such as land surface temperature (LST), actual evapotranspiration (AET), runoff (RO), and wind speed (VS) are not considered direct anthropogenic threats in the conventional sense. Instead, they are incorporated into the model as environmental stress proxies that influence mangrove vulnerability by modulating physiological stress, water availability, and physical disturbance regimes under changing climatic conditions.

**Table 1:** Input values of mangrove habitat quality threats in the InVEST model.

THREAT	MAX_DIST	WEIGHT	DECAY	DESCRIP	UNIT
AET	7.7	0.7	Exponential	Actual evapotranspiration	mm
AOD	4.2	0.4	Linear	Aerosol Optical Depth	Å
DEF	7.6	0.8	Exponential	Climate water deficit	mm
LST	3.5	0.8	Exponential	Land Surface Temperature	^°^C
NO2	3.3	0.3	Exponential	Nitrogen Dioxide	mg/m³
PDSI	5.8	0.7	Linear	Palmer drought severity index	
POP	3.9	1	Linear	Population	People/km^2^
RO	11.2	0.5	Linear	Run off	mm
ROAD	2.5	0.8	Linear	Main road	Km
SO2	2.8	0.3	Exponential	sulfur dioxide	mg/m³
TMMX	7.1	0.8	Exponential	Maximum temperature	^°^C
VDP	5	0.2	Linear	Vapor pressure deficit	Kpa
VS	3.12	0.1	Exponential	Wind speed	m/s
Urban	2.2	1	Linear	Urban Residential Area	ha
ROAD_2	0.12	1	Linear	Rural Roads	Km

The selection and treatment of threats in the InVEST model were based on the specific vulnerability of mangroves in a coastal environment under climate change. While direct anthropogenic threats like aquaculture and industrial mining are not prominent within the strictly protected Khorkhoran area itself, their potential regional pressures are indirectly captured through proxies such as population density (POP), urban expansion (Urban), and road networks (ROAD). Conversely, key environmental variables (including actual evapotranspiration (AET), land surface temperature (LST), wind speed (VS), and runoff (RO)) were explicitly treated as threats because their projected intensification due to climate change (e.g., increased aridity, higher temperatures, more erratic precipitation) directly stresses mangrove physiology and habitat suitability. Although the threat layers in Figs. 3 and 4 show spatial variation (e.g., higher LST or POP near edges), the model inherently accounts for spatial dynamics through the distance-decay functions (MAX_DIST, DECAY), which modulate threat intensity from source pixels into the mangrove habitat. For continuous threats (e.g., AET, LST, AOD, DEF), MAX_DIST was derived from spatial semivariogram analyses, indicating the distance beyond which pixels are considered independent. For location-based threats (e.g., roads, urban areas), MAX_DIST was informed by previous mangrove studies and regional ecology. WEIGHT values (0.1–1) reflect each threat’s relative impact on mangrove health. This approach, while carrying some uncertainty, provides a systematic basis for model parameters.

### Evaluating the accuracy of different methods

To ensure the reliability of our results, the accuracy of the key methodological components was rigorously evaluated using standard quantitative metrics. First, the accuracy of the land use/land cover (LULC) maps, generated using the Random Forest algorithm with variable sampling, was assessed. A confusion matrix was constructed using the 30% hold-out test samples. This evaluation provided key metrics including Overall Accuracy, Kappa Coefficient, Producer’s Accuracy, and User’s Accuracy for each map (2019, 2022, 2024). Second, to identify the optimal model for predicting future habitat quality, ten machine learning algorithms were implemented and compared. Their performance was evaluated using a suite of regression statistics (including the Coefficient of Determination (R²), Mean Absolute Error (MAE), and Root Mean Square Error (RMSE)) calculated on the test data (Table 3). The model with the highest R² and lowest error metrics was selected for the final prediction. Finally, the Otsu thresholding method was employed to objectively classify continuous habitat quality values into binary optimal and sub-optimal classes for consistent temporal analysis.

### Predicting changes in habitat quality using machine learning techniques

The 2029 projection assumes that the relationships between habitat quality and its driving threats observed during 2019–2024 will continue linearly into the near future. To identify the most robust extrapolation model, ten machine learning algorithms (ranging from linear and distance-based methods to ensemble tree and nonlinear models) were evaluated. Model performance was compared using multiple statistical metrics (R², MAE, RMSE) on a test dataset, and the best-performing model was selected to generate the final 2029 habitat quality map.

In the present study, to predict changes in mangrove forest habitat quality in 2029, raster images of habitat quality from 2019 to 2024 were used with Linear, Bayesian, KNN, RF, XGBoost, LGBM, CatBoost, SVR, Neural Network, and Linear Trend machine learning techniques.

**Linear regression: **The LR technique handles a set of records with values of X and Y. The goal of regression is to determine the value of Y, provided that XY is continuous. Regression may be performed using a variety of functions or models, but the most common is the linear function^51^.

**Bayesian machine learning: **Bayesian Machine Learning (BML) is a new approximate dynamic programming approach that fits a set of Bayesian regression models, in reverse order. Each model uses the residual return as the response variable, assuming that optimal actions (or treatments) are taken in subsequent steps, and uses the current history and related actions at that step as auxiliary variables^52^.

**KNN: **The K-Nearest Neighbor classification algorithm is based on supervised learning. Supervised learning can be understood in such a way that a supervisor in supervised learning can also be called a teacher. Supervised learning is a type of learning in which a machine is trained from observer data or labeled data. Then after training, the machine can predict the correct output for any other input. In supervised learning, the input and output data are already provided to the machine, which is called training data or labeled data^53^.

**Random forest algorithm: **Random Forest Algorithm (RF), introduced by Breiman^40^, is an ensemble learning algorithm based on decision trees. It is used for both classification and regression problems. RF collects information by randomly selecting features from each decision tree in the forest. It then uses a majority voting approach for classification or an averaging approach for regression problems to arrive at a final prediction.

**XGBoost: **The Extreme Gradient Boosting Algorithm, proposed by Chen and Guestrin^54^, is a supervised machine learning technique based on the gradient boosting decision tree. This algorithm is a boosting model that uses a sequential grouping technique and combines several base regression trees in a series. The algorithm involves sequentially adding and training new decision trees to adjust the residuals from previous iterations. The estimation values of all these decision trees are then summed to obtain the final estimation result. This algorithm also improves the estimation performance by minimizing the bias in the model and significantly reducing the risk of overfitting the model.

**LightGBM: **The Light Gradient Boosting Machine (LightGBM), proposed by Ke et al.^55^, is an efficient and open-source gradient boosting decision tree-based algorithm used for regression, sorting, and classification problems. Its main distinction from XGBoost is that LightGBM uses a histogram-based algorithm, which results in a faster training process and reduced computational complexity. It can also increase accuracy by reducing training errors. However, this improvement in accuracy comes with the potential downside of increasing the risk of overfitting.

**CatBoost: **The CatBoost algorithm, proposed by Prokhorenkova et al.^56^, is a gradient boosting decision tree algorithm. It is developed to handle classification features more efficiently. In this method, classification features can be replaced with corresponding mean label values. These mean label values are then used as a criterion for node splitting during decision tree construction. This innovative approach effectively minimizes overfitting and accelerates model estimation.

**Support vector regression: **Support vector machines are supervised learning models that analyze data for classification or regression analysis (SVR). Given a training dataset where X is the space of input patterns, in SVR the main goal is to find a function that is characterized by the maximum deviation from the true target values obtained y_i_ for all training data^57^.

**Neural network: **The neural network is a powerful machine learning model that can effectively deal with complex nonlinear relationships and large-scale data. The algorithm integrates advanced evolutionary strategies into neural network training to increase convergence speed, improve solution quality, and enhance generalization capabilities for complex optimization tasks. Through multi-level neural structures and nonlinear activation functions, neural networks can learn and express complex patterns and higher-order features in data to adapt to different data distributions and complex relationships^58,59^.

**Linear trend: **In the context of machine learning and time series analysis, a linear trend denotes a consistent and uniform pattern in the data, where the dependent variable changes at a constant rate over time. Such a trend is typically modeled using simple linear regression, in which time is treated as the independent variable and the observed data as the dependent variable. Despite its simplicity, this model is widely applied as a fundamental tool for identifying long-term trends and generating reliable forecasts^60^.

**Fig. 5:**
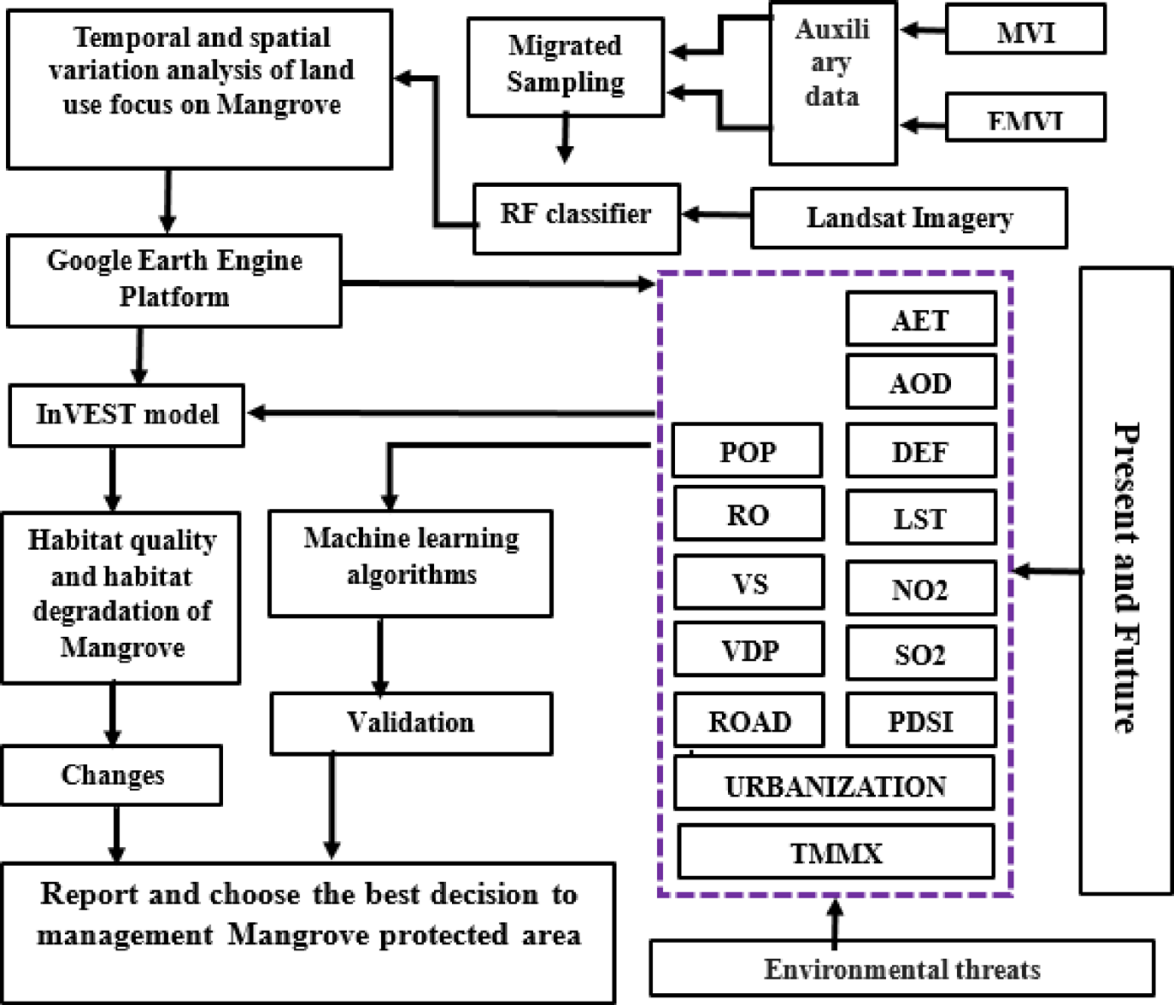
The schematic diagram representation of methodology.

Figure 5 illustrates the schematic diagram of our research methodology.

## Results

### Land use changes

This study assessed the habitat quality in the Khorkhoran mangrove forest using Google Earth Engine (GEE) and the InVEST model. The extent and spatial distribution of land cover/land use (LULC) classes were analyzed for the baseline year (2019) and the current year (2024). The analysis employed a variable sampling method in conjunction with the Random Forest machine learning algorithm.

While earlier studies have estimated and predicted LULC changes using InVEST model plugins, this research directly applied the variable sampling algorithm and Random Forest classification within the GEE framework^61^. Table 2 illustrates the observed LULC changes in the mangrove forests of the study area from 2019 to 2024.

**Table 2:** Land use changes in the study area from 2019 to 2024.

Land use	Area (ha) per year		
	2019	2022	2024
Mangrove	7530.74	7037.65	6546.51
Water bodies	33113.30	32326.00	33741.20
Saline and Barren Lands	17729.90	15439.5	16431.50
Built up	72.52	74.30	96.48
Range and Croplands	1456.42	1263.11	1211.82
Wetlands	8651.03	12413.30	10526.40

As indicated in Table 2, the area of mangrove forests in Khorkhoran decreased by 493.09 hectares between 2019 and 2022, followed by an additional reduction of 491.14 hectares from 2022 to 2024. In total, approximately 984.23 hectares of mangrove forest were lost in the study area from 2019 to 2024, resulting in an average annual loss of around 164 hectares over the six-year period.

### Predicting changes in mangrove forest habitat quality using machine learning techniques

For training each of the aforementioned models, the raster habitat quality layers produced by the InVEST model for the years 2019 (historical data) and 2024 (current data) were used as the base dataset. The 2019 map was considered as training data and the 2024 map for testing the models. All models were implemented and trained using the scikit-learn library in Python. Default or optimized values based on Grid Search were used for the parameters of each model. The predictive performance of the models on the test data (2024) was evaluated using standard metrics, including the Coefficient of Determination (R²), Mean Absolute Error (MAE), Mean Squared Error (MSE), and Root Mean Squared Error (RMSE). These criteria, presented in Table 3, were used to compare the algorithms and select the optimal model for predicting mangrove habitat quality.

**Table 3:** Comparison of zoning accuracy of different machine learning algorithms in predicting habitat quality 2029.

Model	*R* ^2^	MAE	MSE	RMSE	Explained Variance	Max Error
Linear	0.96733	0.036639	0.00273	0.052246	0.967334	0.198812
Bayesian	0.96733	0.036639	0.00273	0.052246	0.967334	0.198814
KNN	0.986793	0.015402	0.001103	0.033219	0.986795	0.247927
RF	0.984814	0.016481	0.001269	0.03562	0.984816	0.227281
LGBM	0.989053	0.014487	0.000915	0.030244	0.989054	0.211814
CatBoost	0.989005	0.014095	0.000919	0.030309	0.989007	0.222697
XGBoost	0.988887	0.014381	0.000929	0.030472	0.98889	0.237267
SVR	0.987764	0.015515	0.001022	0.031974	0.987805	0.209602
Neural Network	0.987981	0.015872	0.001004	0.031689	0.988006	0.194625
Linear Trend	0.998722	0.010333	0.000107	0.010333	1	0.010333

To predict the 2029 habitat quality, the final output was not derived from an ensemble or combination of multiple models. Instead, each model was trained and validated independently on the 2019–2024 habitat quality raster data, and their performance was rigorously compared using the evaluation metrics in Table 3. The selection of the final predictive model was based on a comprehensive evaluation, prioritizing the highest coefficient of determination (R²), the lowest error metrics (MAE, MSE, RMSE), and explained variance. Based on these objective criteria, the Linear Trend model was identified as the unequivocally best performer (R²=0.9987, MAE = 0.0103). Consequently, the future 2029 habitat quality map presented in the results (Figs. 7, 8 and 9) was generated exclusively using this superior Linear Trend model. This selection ensures the most reliable forecast based on the historical trend observed between 2019 and 2024.

On the other hand, to examine the changes in the quality of mangrove forests in these years under study and what will happen to them in the future, determining the threshold levels was performed using the Otsu method to examine the extent of areas with excellent and non-excellent habitat quality (codes 1 and 0) for the years 2019, 2024, and 2029.

As shown in Table 3, the Linear Trend, Neural Network, and SVR models performed better than the other models. Among these three, the Linear Trend method provided the best results in predicting future habitat quality, as illustrated in Fig. 5.

**Fig. 6:**
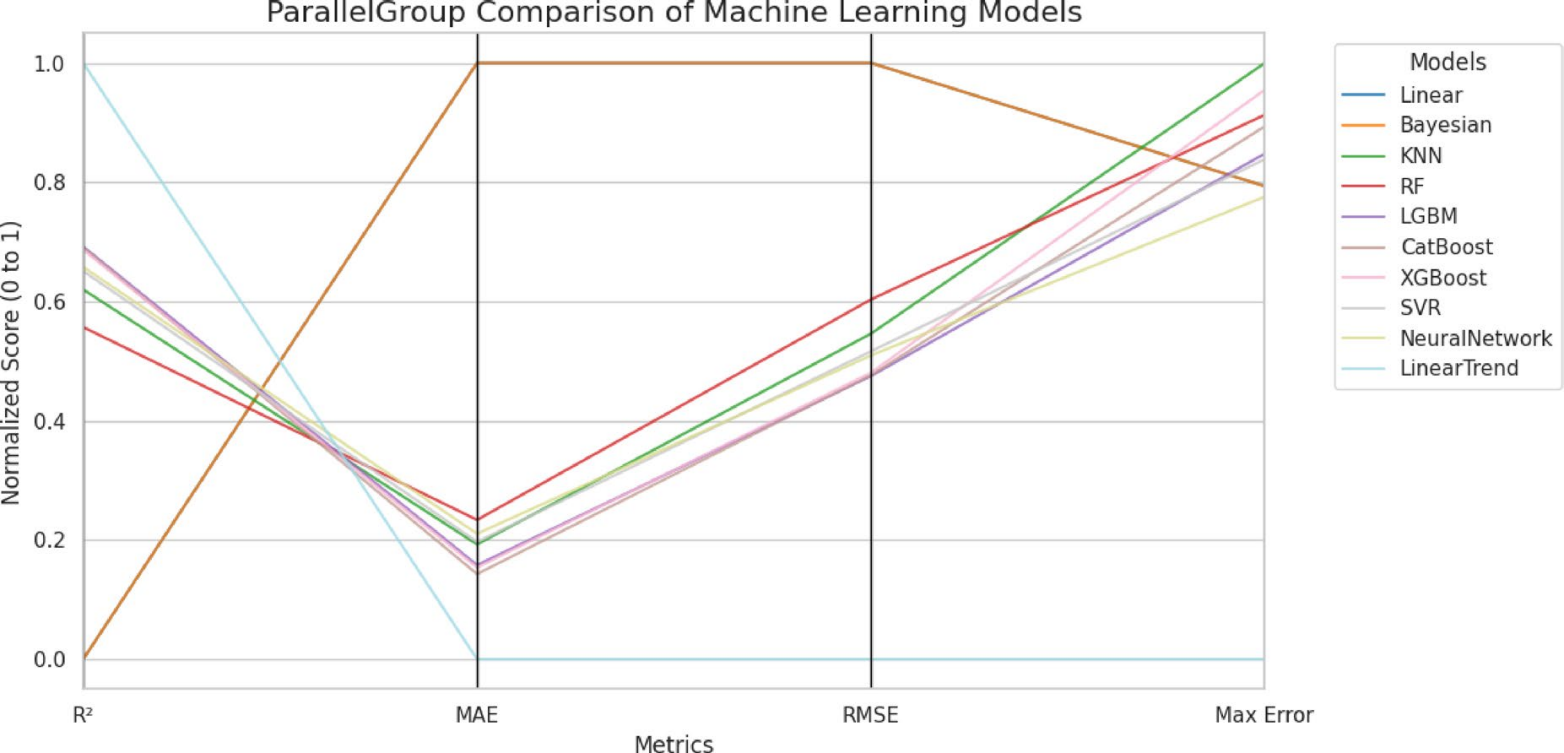
Comparison of different statistics for assessing the accuracy of predicting the quality of mangrove forest habitat with different machine learning techniques.

**Fig. 7:**
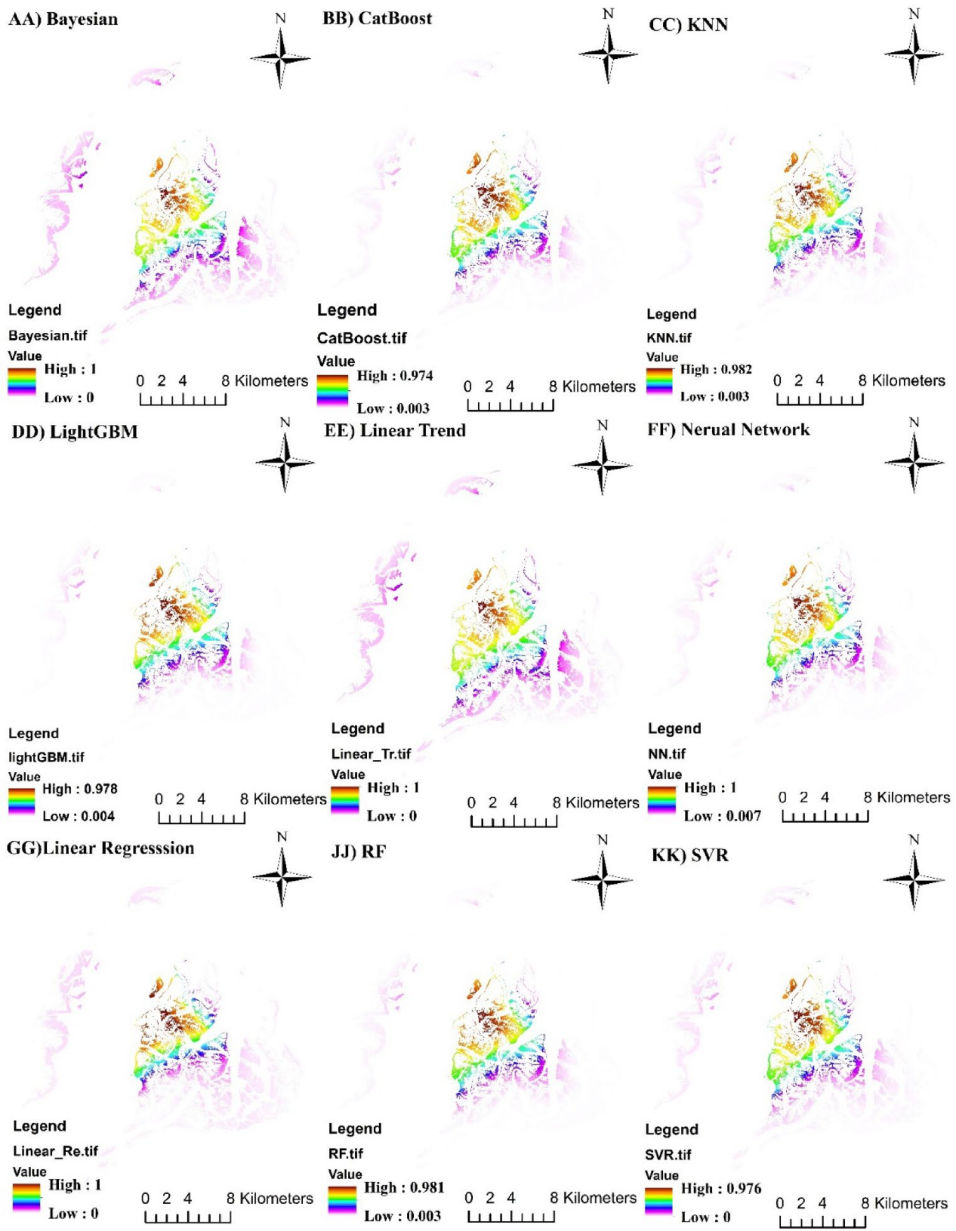

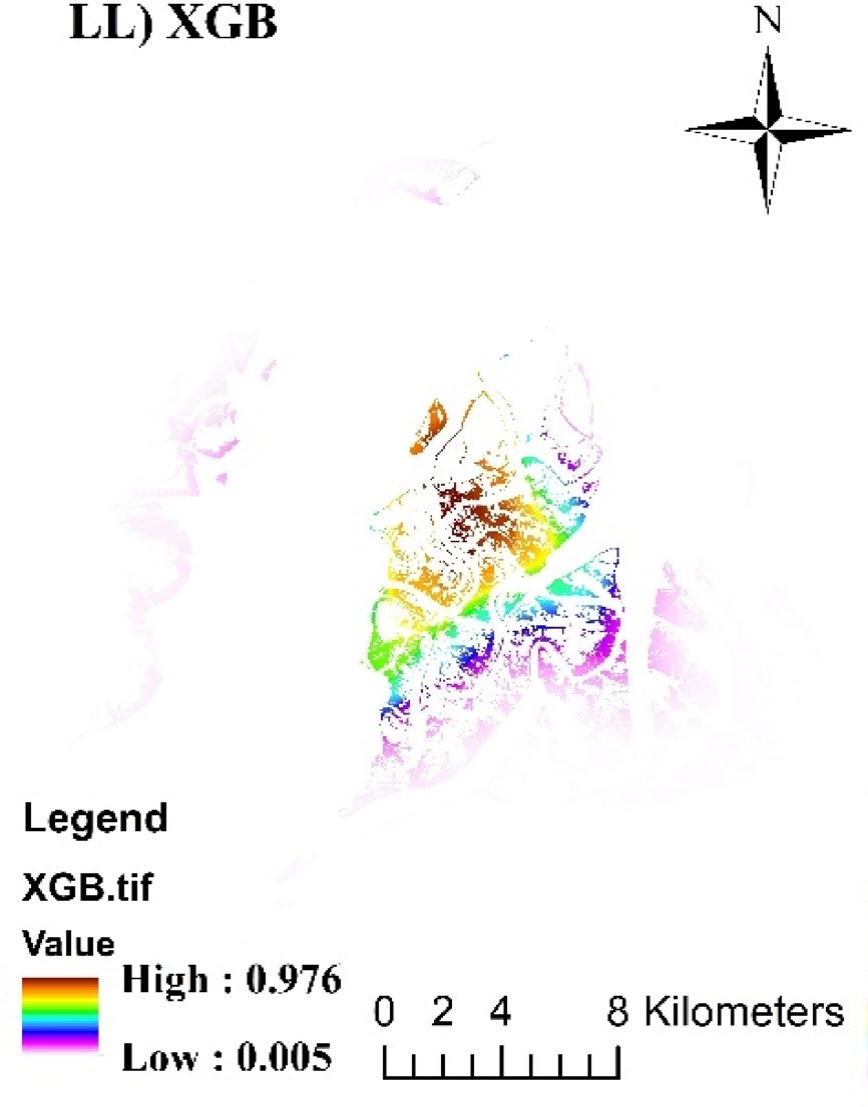
Zoning results of habitat quality prediction with different machine learning algorithms for the year 2029 (Map processing and creation were carried out using ArcMap within ArcGIS version 10.1^32^.

**Fig. 8:**
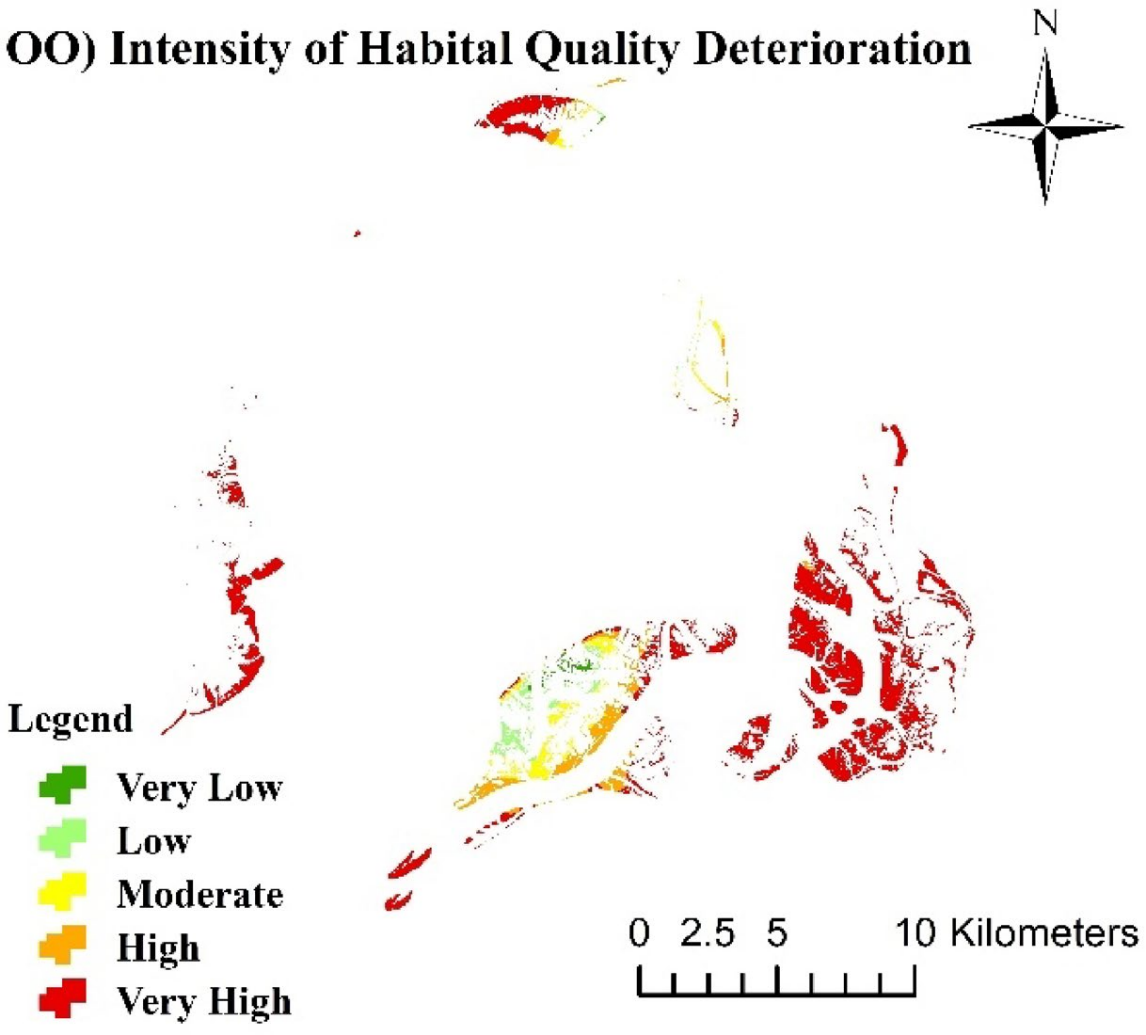
The severity of habitat quality decline in mangrove forests (Map processing and creation were carried out using ArcMap within ArcGIS version 10.1^32^.

Figure 7 also presents the spatial prediction of habitat quality for the year 2029 using machine learning algorithms. Moreover, Fig. 8 shows the severity of habitat quality in the mangrove forests of the study area.

As shown in Figs. 7 and 8, in the future, the very high-quality habitat quality centers will be limited to more inland areas and will be further away from border areas and human communities. Mangrove forests in the outer areas will experience a sharp decline in habitat quality, and areas with moderate habitat quality will also tend to the inner layers, and areas with lower quality will expand further within the mangrove forest areas. In this regard, the maps presented in Fig. 7 also show that the greatest decline in habitat quality occurs from the southwest and south of the study area (from Qeshm Island) towards more inland areas. Habitat quality will also decline from the northwestern parts of the area (Khamir city). The severity of habitat quality decline will be lower in the northeastern areas of the study area, and this decline will gradually become less severe in more central areas. Figure 8 also shows the details of the decline in habitat quality intensity for the year 2029. The area with very high intensity of habitat quality decline will occupy an area of 2188.4 hectares, the area with high intensity of habitat quality decline will occupy an area of 325.9 hectares, the area with medium intensity of habitat quality decline will occupy an area of 180.17 hectares, the area with low intensity of habitat quality decline will occupy an area of 100.69 hectares, and finally the area with very low intensity of habitat quality decline will occupy an area of 27.35 hectares. As can be seen, the largest area of habitat quality decline with very high intensity is more distributed in the outer layers of mangrove forests, and the above figures below shows this well.

**Fig. 9:**
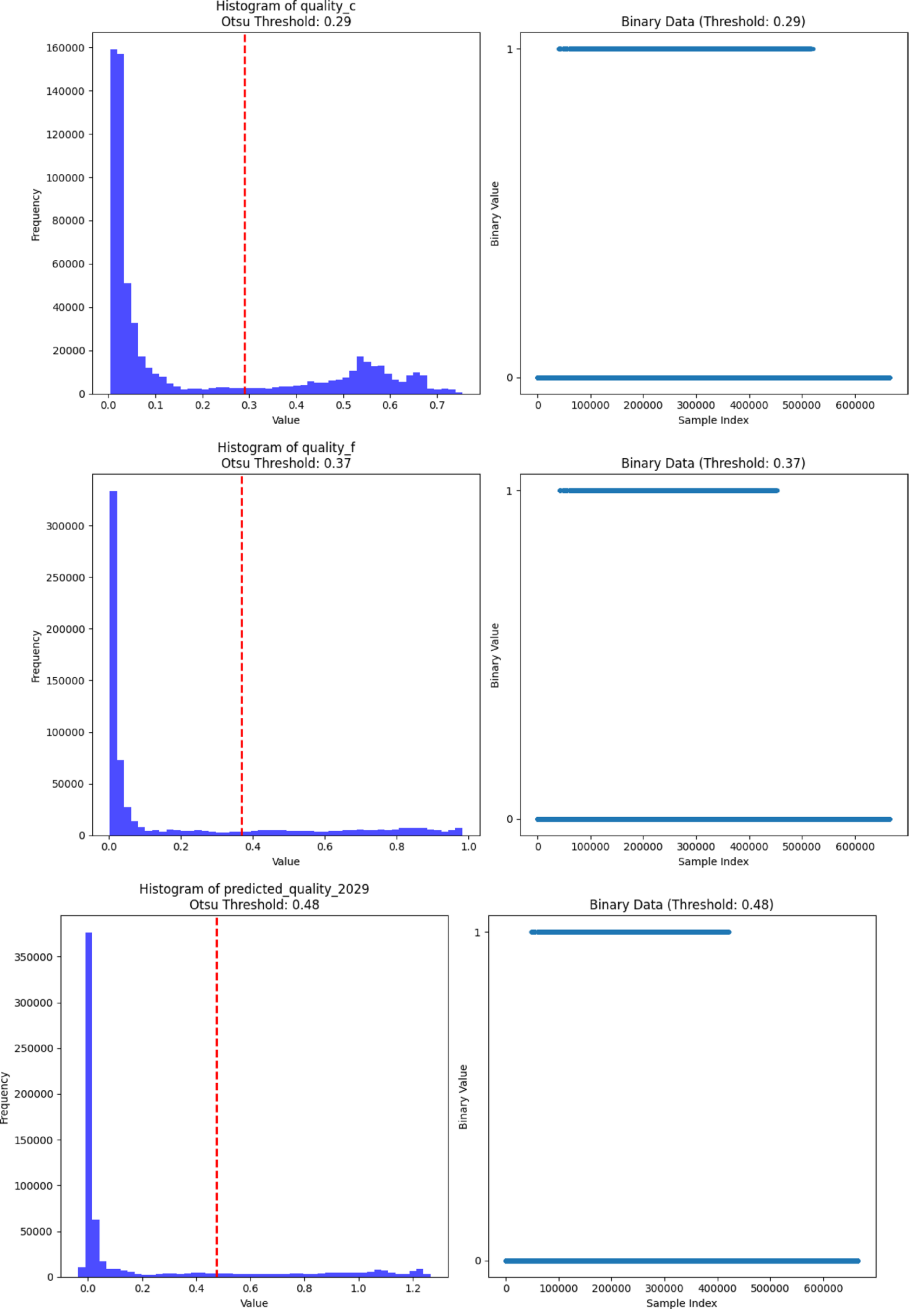
Otsu thresholding diagram for habitat quality in 2019, 2024, and 2029.

Figure 9 also shows the threshold determination using the Otsu method for the changes in mangrove forest quality during the study years and what is expected to happen to them in the future.

The results of Fig. 9 show that areas with sub-optimal habitat quality have shown an increasing trend from 2019 to 2029, and naturally, areas with code 1, or in other words, areas with excellent habitat quality, have shown a decreasing trend, despite the increasing intensity or habitat quality number, which indicates a tendency towards the inner layers of mangrove forests.

### Future changes in mangrove forest degradation

**Fig. 10:**
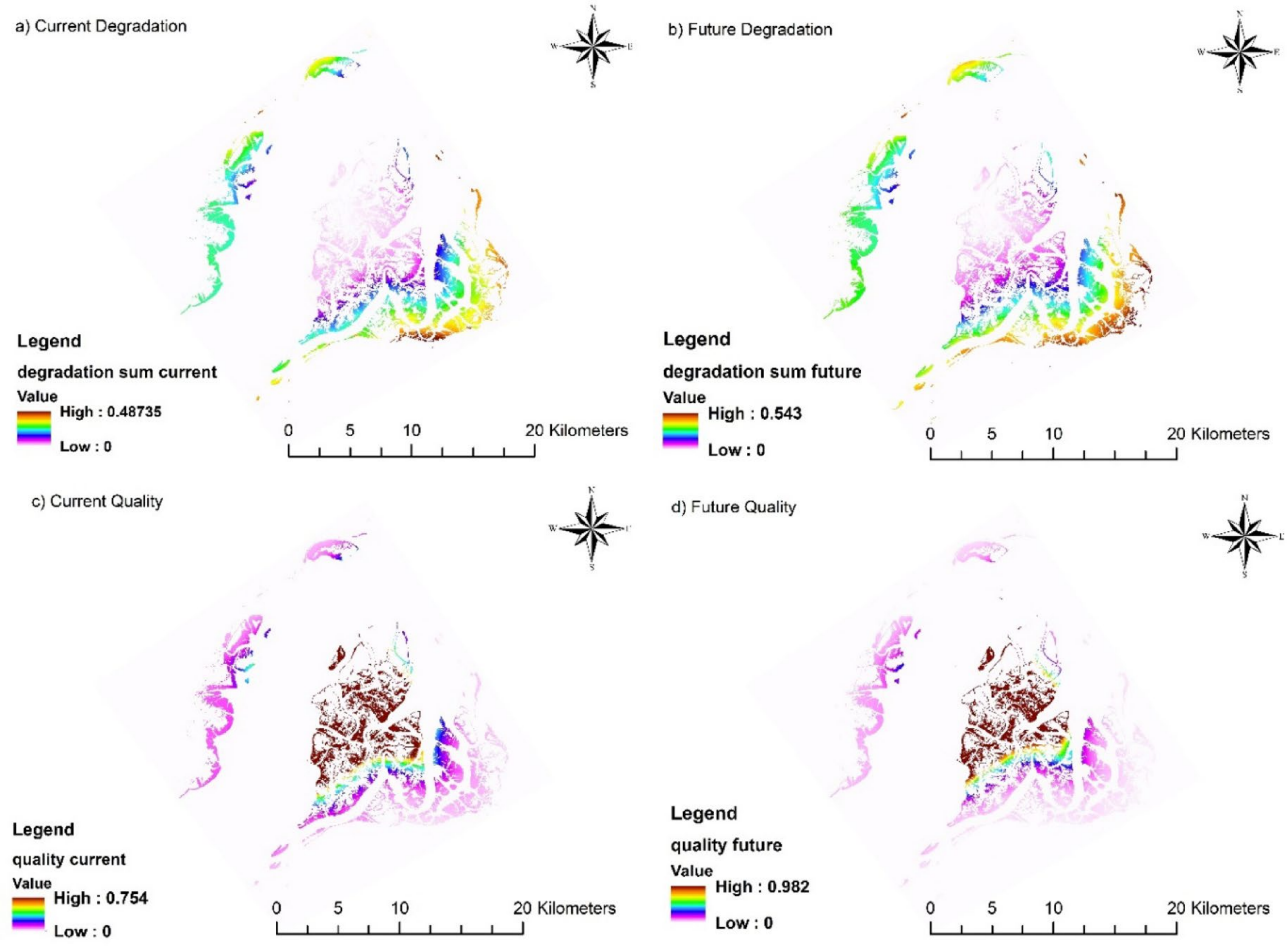
Degradation and habitat quality in the Khorkhoran mangrove forest in current and future conditions (Map processing and creation were carried out using ArcMap within ArcGIS version 10.1^32^.

The results indicate that the most significant mangrove forest degradation is concentrated in the southern and southeastern parts of the Khorkhoran range, with a severely degraded area measuring 534.12 hectares. Surrounding this core zone is a high-degradation layer that spans 1,296.73 hectares. As shown in Fig. 10, this degradation class extends across the southeastern, southern, southwestern, and northern regions of Khorkhoran.

The northern section of the study area primarily falls into the medium-degradation category. Notably, degradation intensity is significantly higher on the side facing Qeshm Island compared to the area near Khamir Port. In contrast, the central mass of mangrove forests, particularly towards the central waters of the study area, exhibits lower degradation intensity.

**Fig. 11:**
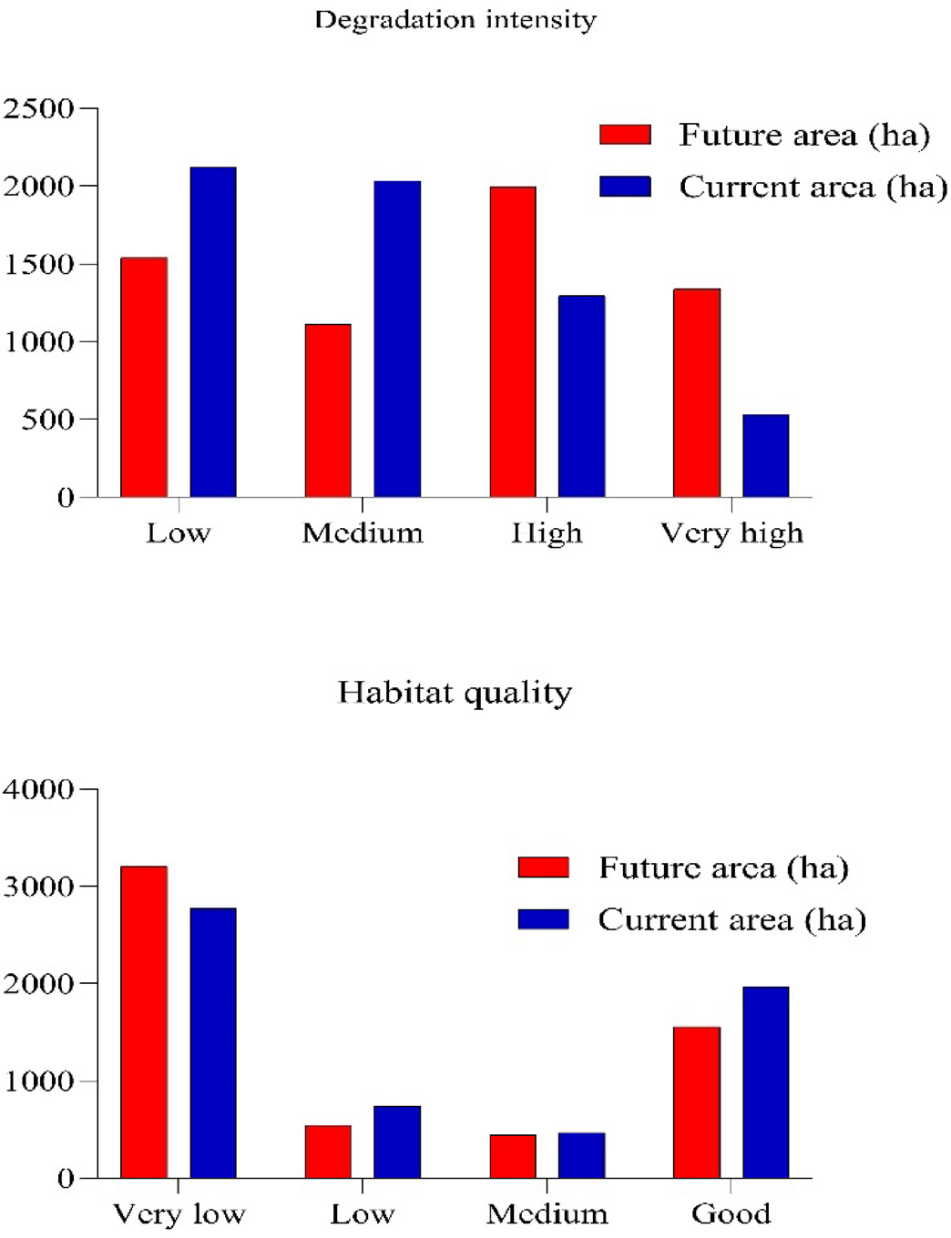
Comparison of present and future levels of degradation intensity and habitat quality in the Khorkhoran mangrove forest.

The spatial patterns of degradation and habitat quality decline shown in Figs. 7, 8, 9, 10 and 11 result from the integrated impact of both anthropogenic pressures and intensified climatic stressors, as modeled in InVEST. The pronounced degradation in southern areas adjacent to Qeshm Island is primarily driven by proximate human threats, including higher population density (POP), urban expansion (Urban), and road networks (ROAD), which directly fragment and pressure the mangrove fringe. In contrast, the widespread decline in habitat quality across broader areas, including more inland sections, is strongly linked to the intensified climatic threat layers, particularly increased land surface temperature (LST), vapor pressure deficit (VDP), and drought severity (PDSI). While direct point-source industrial or aquaculture threats are not dominant within this protected area, their regional influence and the overarching stress from climate change are captured through these climate-derived variables and the human footprint proxies. Therefore, the projected degradation represents a combined stress scenario where direct human encroachment exacerbates the ecosystem’s vulnerability to background climatic extremes.

The results suggest that the highest levels of degradation are expected to persist in the southern areas, with additional northern regions projected to transition into the high-degradation class. The future extent of mangrove forest degradation in Khorkhoran is predicted to cover 1,341.65 hectares. Moreover, within the inner regions of the southern mangrove habitat, multiple degradation layers are anticipated, extending across both the southern and northern parts of the study area. The size of these highly degraded zones is projected to reach 1,994.94 hectares, replacing areas currently classified as medium-degradation.

In summary, regions in the northern part of the study area, which are currently experiencing moderate degradation, are expected to shift into the high-degradation class in the future. Consequently, the medium-degradation class is projected to decrease to 1,111.36 hectares. As illustrated in Fig. 10, low-degradation areas will also become more limited, reducing to 1,538.33 hectares compared to current conditions.

Figure 11 illustrates that the area classified as high-degradation is expected to expand significantly in the future, increasing by approximately 698.22 hectares compared to current conditions. In contrast, the medium-degradation class will decrease by 923.2 hectares, while the low-degradation class will shrink by 582.54 hectares. The intensity of degradation is projected to exceed current levels, with the maximum intensity rising from 0.48 (current) to 0.54 (future).

For the very high-degradation class, the intensity range is anticipated to increase from 0.29 to 0.48 (current) to 0.31–0.54 (future). Similarly, the high-degradation class will shift from a range of 0.21–0.29 to 0.21–0.31, while the medium-degradation class will remain steady at 0.11–0.21.

In terms of habitat quality, the results indicate that the central areas of the study site currently exhibit the highest habitat quality, classified as good habitat quality, covering an area of 1,966.7 hectares. This is followed by the medium-quality class, which spans 469.83 hectares and is distributed in the outer layer extending from the center to the southern parts of the mangrove area. The low-quality habitat class occupies 745.64 hectares, while the very low-quality class is concentrated in the marginal areas, covering 2,776.51 hectares.

For future conditions, the central areas of the Khorkhoran mangrove forests are expected to retain the highest habitat quality, although the area will decrease to 1,757.2 hectares. The medium-quality class will remain in the outer layers, covering 443.72 hectares. Meanwhile, the low-quality and very low-quality habitat classes are projected to expand, occupying 546.1 hectares and 3,211.85 hectares, respectively.

It is important to note that regions with lower habitat quality correspond to areas with higher degradation intensity, as previously observed ^62^. Currently, the highest proportions of degradation are found in the low and medium degradation classes, accounting for 35.43% and 33.99%, respectively. Under future conditions, the highest proportions are expected to shift to the high (33.33%) and low (25.7%) degradation classes. The most significant changes are projected for the medium degradation class, which will decrease by 15.42%, and the very high degradation class, which will increase by 13.49%.

In terms of habitat quality, the very low-quality class currently occupies the largest share of the Khorkhoran mangrove forest habitat at 46.6%, followed by the good-quality class at 33.01%. For future conditions, the very low-quality class is expected to increase to 53.9%, while the good-quality class will decrease to 29.49%. This reflects a 3.7% expansion in the very low-quality class and a 3.51% reduction in the good-quality class.

The medium-quality class is projected to undergo minor changes, with only a slight increase of approximately 0.44%. However, the low-quality habitat class is expected to shrink by 3.35% compared to current conditions.

## Discussion

The results are interpreted within the study’s methodological framework and underlying assumptions. Land cover classification was based on remote sensing and validated reference data rather than field surveys, and some environmental variables were used as proxies, introducing uncertainty in absolute degradation and habitat quality values. Nevertheless, the relative spatial patterns and temporal trends (including high-risk zones in the south and the decline in high-quality habitats) are robust, as they stem from a consistent model application across time and space, supporting reliable comparative risk assessment and conservation prioritization.

In the current study, the effects of soil characteristics, climatic variables, land cover changes^63,64^, and human activities were integrated into the habitat quality assessment model. Unlike traditional approaches, the InVEST model extension was not employed to estimate land use changes. Instead, an innovative method was implemented using samples from 2019 to 2024, along with the Random Forest machine learning classification algorithm, within the implemented within the Google Earth Engine (GEE). A significant advantage of this research was the incorporation of diverse satellite imagery as inputs to the InVEST model. Also, various machine learning models were studied to assess habitat quality.

The results revealed that the average intensity of degradation under current conditions was 0.01, which is projected to increase to 0.02 in future conditions. This represents a doubling of the degradation intensity of mangrove forests in the Khorkhoran region over a six-year period. Furthermore, habitat quality is expected to decline from 0.019 under current conditions to 0.017 in future conditions, indicating a decrease in habitat quality across the study area.

Similar to the results of Admasu et al.^8^, this study indicates a shift in habitat quality from areas of excellent quality to those of lower quality. In the referenced research, the area of high-quality habitats in the studied watershed decreased from approximately 95% in 1985 to 23.28% in 2022. Concurrently, areas with low habitat quality expanded from 4.46% to nearly 76% of the studied region. In the current study, over a short-term period of six years, the proportion of the good-quality habitat class decreased from about 33% to approximately 29%, while the proportion of the low-quality class increased from about 46% to 54% of the study area. Additionally, the results of the current study align with those of Li et al.^62^, indicating the reduction in habitat quality.

The expansion of low habitat quality in the Khorkhoran region, coupled with the loss of natural habitat, could have significant impacts on the ecosystem and the well-being of the local population. It is crucial to recognize that the livelihoods of local residents depend on mangrove tourism and fishing, both of which are directly linked to the health of the mangrove ecosystem. The degradation of these habitats could adversely affect their daily lives and overall quality of life.

This study integrated spatial data, including Land Use Land Cover (LULC) maps, following the methodologies used by Zafar et al.^14^. By incorporating mangrove vegetation indices (MVI) and land use change maps, developed using the variable sampling method and Random Forest classification, the study revealed a positive relationship between healthy vegetation away from human settlements and habitat quality. It also demonstrated that proximity to human activities correlates with a higher intensity of habitat degradation.

These results are consistent with previous studies, such as those by Nagendra et al.^65^, Baral et al.^66^, and Berta Aneseyee et al.^50^, which reported that intact vegetation and reduced human interference contribute to better habitat quality and lower degradation levels.

In this study, areas where mangrove forest cover has fragmented (shifting from dense stands to smaller, isolated patches closer to human threat sources) exhibited significant habitat quality loss. Conversely, the central regions of the study area experienced relatively lower degradation over time. However, the rings of habitat quality loss are expanding outward from the edges toward the core of the mangrove forest. This shows the need for monitoring and managing effective buffer zones surrounding protected areas, particularly where habitat quality is diminishing.

The reported average degradation (0.01–0.02) and habitat quality (0.019–0.017) appear low but reflect the model’s relative scaling, not absolute ecological condition. These InVEST-generated indices depend on parameter choices (half-saturation constant, threat weights, influence distances) and indicate spatial comparisons rather than absolute fitness. The study area’s configuration also influences these magnitudes. With threat sources concentrated at the periphery and distance-decay functions limiting interior influence, large central areas show minimal degradation, lowering overall averages. Despite small absolute values, proportional changes are ecologically meaningful: degradation doubling over six years signals accelerating pressure, while the 3.52% reduction in “good” habitat area (33.01% to 29.49%) provides an intuitive metric of habitat loss. Interpretation should therefore focus on relative spatial patterns (high-risk southern zones), temporal trends (degradation intensification), and proportional class changes, rather than absolute numerical values.

The observed pattern of severe decline in the outer mangrove layers is a direct outcome of the InVEST model’s structure, which incorporates a distance-decay function for threats. This function assumes that the impact of a threat source (e.g., urban areas, roads) diminishes with increasing distance. Consequently, pixels closest to these threat sources, predominantly located at the forest edge, experience the highest degradation. This aligns well with the ecological reality of edge effects, where habitats near boundaries are more vulnerable to external disturbances. However, as the reviewer notes, the model simplifies reality by applying this decay uniformly from defined threat sources. It does not dynamically simulate the gradual internal fragmentation or penetration of threats (e.g., illegal logging trails, pollution plumes) that can originate from the edge and propagate inward over time. Therefore, while the model reliably identifies zones of highest initial risk at the periphery, the predicted intensity and exact spatial gradient of decline towards the interior should be interpreted as a conservative projection of pressure from external sources, acknowledging that internal degradation processes are not explicitly captured.

The severe decline observed in the outer mangrove layers reflects the InVEST model’s distance-decay function, where threats (e.g., urban areas, roads) have diminishing impact with distance. As a result, forest-edge pixels experience the highest degradation, consistent with ecological edge effects. However, the model applies this decay uniformly and does not simulate gradual internal penetration of threats. Thus, while it reliably identifies high-risk peripheral zones, the predicted intensity and gradient toward the interior should be seen as a conservative estimate, as internal degradation processes are not explicitly modeled.

Human activities in the region, such as the expansion of settlements near mangrove forests and urbanization^67,68^, pose significant threats to habitat quality^69,70^. The southern parts of the study area, where the distance between human settlements and mangrove forests is minimal, exhibit higher levels of habitat degradation and a more pronounced reduction in mangrove habitat quality compared to the northern regions. As noted by Yu et al.^70^, human activities tend to concentrate in plains and floodplains with low slope indices. Given that the present study area is predominantly flat with minimal slope variations, it is particularly vulnerable to these impacts.

The spatial degradation maps generated in this study identified zones of high-intensity degradation and at-risk areas requiring rehabilitation, providing a valuable tool for targeted conservation planning. Although the InVEST model focuses on binary habitat classifications (habitat versus non-habitat (1 or 0)) and does not account for factors such as habitat size, quality, or functional conditions, it remains a powerful tool for identifying conservation priorities. This aligns with results by Terrado et al.^71^ and Fang et al.^72^, who proved the model’s effectiveness in ecosystem service modeling for conservation planning.

One of the key features of the InVEST model is its ability to assess habitat sensitivity to various threats and estimate the relative impact of specific threats compared to others. Researchers such as Yohannes et al.^73^ and Yu et al.^70^ have praised InVEST modeling as a valuable tool that provide perceptive perspectives for decision-makers. For instance, Yu et al.^70^ utilized the InVEST model to analyze habitat quality across the Qinghai Plateau of Tibet from 2000 to 2020, incorporating various information layers such as average annual precipitation, gross domestic product, population density, soil type, slope, vegetation index, Shannon’s diversity index, density of biological hotspots, average annual temperature, and a digital elevation model. In their study, elevation and topography were identified as significant factors affecting habitat quality.

In contrast, the present research focused on a low-altitude marine and coastal habitat, where different variables were more pertinent for analysis. The study utilized variables such as soil texture, dust, population density, human construction index, mangrove area identification indicators, maximum temperature, soil moisture deficiency, runoff, air pollutants, ground surface temperature, wind speed, changes in water vapor pressure, and access roads to estimate habitat quality and degradation intensity. Unlike regions with diverse elevations, where slope and topography play crucial roles, the flat terrain of the study area exhibited negligible changes in slope, rendering these variables less significant. Furthermore, slope is not a dynamic variable and does not substantially affect habitat quality in wetlands, as noted in similar studies, such as Wang et al.^12^.

Roy et al.^74^ studied and predicted the replanting potential of mangrove forests in the Arabian Peninsula using correlations between 8 variables: altitude above sea level, soil acidity, average rainfall, average and minimum land surface temperature, soil salinity, soil texture, and distance from urban areas using machine learning techniques of Random Forest, XGB, SVM, and NB (Naive Bayes). They considered the Random Forest algorithm to be the most efficient predictor for the research topic. They also considered the SVM algorithm to be appropriate. In the current study, variables such as the variables mentioned were used to estimate the habitat quality of mangrove forests in the Persian Gulf region from the Iranian waters side, and after estimating the habitat quality based on Sentinel 2 images and GEE data in 2019 and 2024, the habitat quality was predicted for 2029 using machine learning techniques. In this study, 9 machine learning methods and the linear trend method were used to predict changes in habitat quality. Among the machine learning techniques, the NN and SVR methods provided better results and in this regard are similar to the results of the aforementioned study. The results showed that the linear trend method performed better than the two machine learning algorithms, artificial neural network and SVR.

Meanwhile, the use of artificial neural network learning technique to predict changes in mangrove habitat quality in the Persian Gulf region in the current study was identified as a good model for predicting future changes in mangrove forests, which is consistent with the results of Farzanmanesh et al. (2025), Saoum and Sarkar (2024). Despite the increase in the area of mangrove forests in the aforementioned study, a decrease in the quality of mangrove forest habitat was observed for the Khurkhoran area from 2019 to 2024, and this trend will continue for 2029. As the results showed, despite the increase in the intensity of habitat quality due to the internalization and retreat of high-quality forests, the area of excellent and high-quality mangrove areas is decreasing according to the Otsu threshold limits. On the other hand, the results of Mondal et al.^24^ using the habitat quality index of the INVEST model and ANN machine learning for changes in habitat quality degradation of the Sundarbans region forests (between India and Bangladesh) showed that the habitat quality and the rate of its degradation differ significantly among different areas of the Sundarbans mangrove forest, which is also consistent with the results of the present study.

This study primarily investigated dynamic variables (excluding soil texture) whose temporal changes influence habitat quality and degradation intensity. The exclusion of static variables like slope aligns with the unique characteristics of the mangrove ecosystem, where dynamic environmental and anthropogenic factors have a more direct and measurable impact on habitat conditions.

### Interpretation of results within modeling assumptions

The spatial patterns presented should be interpreted as model-based outcomes conditioned on our methodological choices, rather than direct representations of actual ecological processes. First, variables such as LST, AET, runoff, and wind speed are environmental stress proxies modulating mangrove physiological stress under climate change, not direct anthropogenic threats. Their impact magnitudes indicate relative stress rather than absolute degradation. Second, while southern degradation hotspots spatially associate with human infrastructure, the model’s distance-decay structure inherently assigns higher degradation near threat sources. This aligns with edge effects but does not capture complex degradation pathways like internal fragmentation or pollution plumes. Third, due to data limitations, several human pressures (aquaculture, tourism intensity, boat traffic, oil pollution) were not explicitly included. Their absence means degradation estimates may underrepresent the full spectrum of anthropogenic stressors. Fourth, our LULC classification represents an operational remote-sensing distinction rather than strict ecological separation. Classes like “wetlands” encompass both mudflats and open water, appropriate for InVEST’s binary framework but not capturing finer ecological gradients. Finally, 2029 projections assume temporal continuity in driving factors. Despite excellent statistical performance (R²=0.9987), these should be viewed as scenario-based forecasts under trend persistence, not deterministic predictions. Given these considerations, relative spatial patterns and temporal trends (particularly high-risk southern zones) are robust for conservation prioritization. However, absolute degradation values should be interpreted with appropriate caution.

## Conclusion

This study developed and applied an integrated framework combining Google Earth Engine (GEE), the InVEST model, and machine learning to analyze and predict spatial variations in habitat quality and degradation within the Khorkhoran mangrove protected area. The results demonstrate a clear and concerning trajectory of ecosystem decline. The main factors behind the decline in the area of Hara (mangrove) forests in the Khorkhoran region are both human and natural causes, with human activities having the greatest impact. Due to rising unemployment, poverty, and weak management, the area has been exploited beyond its capacity. Issues such as increased fuel smuggling, oil pollution, and land-use changes have also contributed to environmental problems in the region. Our analysis revealed that, under current conditions, 35.43% of the mangrove area faces low-intensity degradation, while future projections indicate a significant shift, with 33.33% of the area expected to transition to high-intensity degradation, particularly in the southern regions near Qeshm Island. The importance of the obtained results lies in their direct quantification of the mounting pressures on a Mangrove coastal ecosystem. The projected 3.52% decrease in “good and suitable” habitat quality, coupled with the expansion of human activity indices by several square kilometers, emphasizes the tangible threat posed by the synergistic effects of environmental change and anthropogenic pressure. The superior performance of the Linear Trend, Neural Network, and SVR models in predicting habitat quality provides a reliable tool for forecasting future ecological states, which is a notable advancement for proactive conservation. In comparison with previous studies, our results are consistent with global trends of mangrove habitat degradation but offer a novel, high-resolution perspective for the Persian Gulf region. The innovative integration of multi-temporal satellite-derived threat layers) such as population density, LST, and drought severity (directly into the InVEST model within the GEE platform, represents a significant methodological advancement over traditional approaches. This study confirms the established principle that proximity to human activities accelerates habitat degradation, yet it does so with a uniquely comprehensive and dynamic set of variables tailored to a flat, coastal desert environment. The practical applications of this research are immediate and vital for coastal management. The generated maps of degradation intensity and habitat quality pinpoint specific high-risk zones, enabling targeted conservation actions, such as establishing enforced buffer zones in the southern and northwestern parts of the forest. These results can directly inform land-use planning, guide the allocation of conservation resources, and support the development of policies that regulate urban expansion and other detrimental activities in the mangrove periphery. A primary limitation of this study is the inherent constraint of the InVEST model, which utilizes a binary habitat suitability classification and does not account for finer-scale ecological parameters such as species composition or patch connectivity. It should also be acknowledged that due to data limitations, some potential human-induced pressures (such as aquaculture development, industrial activities, or tourism intensity) were not explicitly included as threat layers in the model. Their possible effects, however, may be indirectly represented through proxies like population density and urban expansion. Furthermore, the accuracy of future projections is contingent upon the representativeness of the current threat trends continuing linearly. For future research, we recommend integrating higher-resolution ecological data, such as field-measured biodiversity indices, to validate and refine the model outputs. Investigating the socio-economic drivers behind the identified threats and exploring the efficacy of specific community-based conservation incentives would be a valuable complementary study. Additionally, applying this integrated GEE-InVEST-Machine Learning framework to other critical mangrove ecosystems in the region would allow for comparative analysis and broader regional conservation strategies. In conclusion, this research provides compelling evidence of the ongoing degradation of the Khorkhoran mangroves and delivers a powerful, replicable tool for its monitoring. The results unequivocally indicate the urgent need for strategic intervention to mitigate human impacts and preserve these invaluable ecosystems for biodiversity, climate resilience, and the livelihoods of local communities.

## Supplementary Information

Below is the link to the electronic supplementary material.

Supplementary Material 1

## Data Availability

All data generated or analyzed during this study are included in this published article.
