# Supplementary material for: Integrating InVEST and machine learning to model mangrove habitat degradation trend in in Northern Persian Gulf, Iran
Source: Sci Rep. 2026 Apr 21;16:18563. doi: 10.1038/s41598-026-47305-z (PMC13269540; doi:10.1038/s41598-026-47305-z)
Supplement: Supplementary file 1 — Supplementary Material 1 [file 41598_2026_47305_MOESM1_ESM.docx]

**Appendix:**

The abbreviations and terms used in this study are summarized in Table S1.

Table S1. List of abbreviations and definitions used in this study.

| Abbreviation | Full Form |
| --- | --- |
| AET | Actual Evapotranspiration |
| AOD | Aerosol Optical Depth |
| BML | Bayesian Machine Learning |
| CatBoost | Categorical Boosting |
| CWD | Climate Water Deficit |
| EMVI | Enhanced Mangrove Vegetation Index |
| ESA | European Space Agency |
| GEE | Google Earth Engine |
| InVEST | Integrated Valuation of Ecosystem Services and Trade-offs |
| KNN | K-Nearest Neighbors |
| LGBM | Light Gradient Boosting Machine |
| LR | Linear Regression |
| LST | Land Surface Temperature |
| LULC | Land Use Land Cover |
| MAE | Mean Absolute Error |
| MSE | Mean Squared Error |
| MVI | Mangrove Vegetation Index |
| NDVI | Normalized Difference Vegetation Index |
| NIR | Near-Infrared |
| NN | Neural Network |
| NO₂ | Nitrogen Dioxide |
| PDSI | Palmer Drought Severity Index |
| POP | Population Density |
| RF | Random Forest |
| RMSE | Root Mean Squared Error |
| RO | Runoff |
| SAD | Spectral Angle Distance |
| SO₂ | Sulfur Dioxide |
| SWIR1 | Shortwave Infrared 1 |
| SWIR2 | Shortwave Infrared 2 |
| SVR | Support Vector Regression |
| TMMX | Maximum Temperature |
| VPD | Vapor Pressure Deficit |
| WS | Wind Speed |
| XGBoost | Extreme Gradient Boosting |
